# Human iPSC-derived neurons stimulated with IFNγ present HLA class I-restricted antigens to cytotoxic CD8^+^ T cells

**DOI:** 10.1186/s12974-026-03915-y

**Published:** 2026-06-17

**Authors:** Benjamin DS Clarkson, Susanna Pucci, Brittany L Overlee, Ramila Barun Shrestha, Kiran K Mangalaparthi, Remya Raja, Pei Shang, Marion Curtis, Akhilesh Pandey, Charles L Howe

**Affiliations:** 1 Translational Neuroimmunology Laboratory, Mayo Clinic, Guggenheim 1542C; 200 First St SW, Rochester, MN 55905 USA; 2 Department of Laboratory Medicine and Pathology, Mayo Clinic, Rochester, MN USA; 3 Center for Multiple Sclerosis and Autoimmune Neurology, Mayo Clinic, Rochester, MN USA; 4 Department of Immunology, Mayo Clinic, Phoenix, AZ USA; 5 Mayo Clinic Comprehensive Cancer Center, Mayo Clinic, Phoenix, AZ USA; 6 Manipal Academy of Higher Education, Manipal, Karnataka India; 7 Center for Individualized Medicine, Mayo Clinic, Rochester, MN USA; 8 Division of Experimental Neurology, Mayo Clinic, Rochester, MN USA; 9 Department of Neurology, Mayo Clinic, Rochester, MN USA; 10 Department of Immunology, Mayo Clinic, Rochester, MN USA

**Keywords:** Interferon-γ (IFNγ), HLA class I, Antigen presentation, Immunopeptidomics, Induced pluripotent stem cells, Neural organoid, Neuroinflammation, CD8^+^ T cells

## Abstract

Multiple sclerosis lesions are dominated by clonally expanded CD8^+^ T cells within an IFNγ-rich inflammatory microenvironment and neurons may be targets of these effector cells. However, the peptide antigens that CD8^+^ T cells recognize on neurons are largely undefined. Neurons constitutively express low levels of HLA class I, and whether inflamed human neurons are competent to present a class I ligandome, what that ligandome contains, and whether presentation has functional consequences for autoreactive CD8^+^ T cells remain open questions. Here we combine human iPSC-derived neural aggregates (HNAs), HLA class I immunoprecipitation coupled to LC-MS/MS immunopeptidomics, and microfluidic co-culture assays to map IFNγ-induced HLA class I presentation by neurons and to test antigen-specific cytotoxicity. IFNγ stimulation induced HLA class I upregulation in HNAs and enabled recovery of a canonical 8-12-mer class I ligandome enriched for 9-mers. Neuron-restricted expression of a synapsin-driven polyepitope cassette yielded presentation of defined exogenous 9-mer peptides on donor HLA class I molecules and, in the presence of IFNγ, elicited activation of autologous antigen-specific CD8^+^ T cells and antigen-dependent neurite injury. Across four donors, comparative immunopeptidomics identified IFNγ-associated neural peptide repertoires that were distinct from those of matched fibroblasts and enriched for predicted HLA-B binding peptides. β2-microglobulin deletion ablated peptide recovery, and neuron-restricted reconstitution enabled identification of candidate neuron-derived peptides, including recurrent neurofilament light (NEFL)-derived peptides detected across donors. Together, these findings establish a human iPSC-derived platform for studying inflammatory neuronal HLA class I antigen presentation and antigen-dependent CD8^+^ T cell engagement.

**Supplementary Information:**

The online version contains supplementary material available at 10.1186/s12974-026-03915-y.

## Introduction

Neuroinflammatory diseases of the central nervous system (CNS) are characterized by immune activation in close proximity to neurons and axons, raising the possibility of direct immune cell-neuron interactions that contribute to tissue injury [1]. In multiple sclerosis (MS), long-term disability correlates most strongly with gray matter pathology and neuroaxonal loss rather than lesion counts or relapse activity [2, 3]. Within active MS lesions, MHC class I-restricted CD8^+^ T cells predominate, outnumbering CD4^+^ T cells by an order of magnitude [4], and clonally expanded CD8^+^ populations persist across brain, CSF, and blood compartments, consistent with ongoing recognition of specific antigenic targets [4, 5]. Moreover, CD8^+^ T cell density within lesions correlates with active axonal injury [6], and lesion evolution and axonal injury track with clinical progression and disability accrual [7, 8]. Critically, CD8^+^ T cells are implicated in the pathogenesis, propagation, and/or progression of numerous CNS diseases, including Alzheimer’s disease, tauopathies, and ALS [9–14].

Cytotoxic CD8^+^ T cells mediate antigen-specific damage when target cells display peptides on human leukocyte antigen (HLA) class I molecules. Interferon-γ (IFNγ) is a key mediator of neuroinflammation that induces the antigen-processing machinery and HLA class I expression, potentially rendering neurons and axons more immunologically visible within inflamed microenvironments [1]. We previously showed that IFNγ drives retrograde induction of HLA class I molecules and antigen presentation machinery in human induced pluripotent stem cell(iPSC)-derived neurons [15], and that CD8^+^ T cells recognizing neuron-restricted antigens can injure axons in a model of demyelination [16]. Together, these findings support the premise that inflammatory cues can enable neuronal and axonal antigen presentation capable of engaging antigen-specific CD8^+^ T cells under defined experimental conditions, consistent with broader evidence that cytotoxic T lymphocytes participate in autoimmune and degenerative CNS diseases.

Despite evidence that inflammation renders neurons and axons susceptible to antigen-specific CD8^+^ T cell attack, the naturally presented peptide repertoire displayed by human neurons under inflammatory conditions remains poorly defined. Our prior work emphasized that neuron-specific autoantigens in MS remain unknown and may be difficult to detect using conventional approaches that prioritize dominant immune specificities [16]. In addition, inter-individual variation in HLA haplotypes raises the possibility that neuronal HLA class I ligandomes and the epitopes available for CD8^+^ T cell recognition vary substantially across individuals [1]. This point is underscored by evidence for tissue-specific HLA allotype expression/usage [17], locus-specific promoter differences in interferon-mediated induction of HLA class I genes [18], and genetic associations between specific HLA class I alleles and neuroinflammatory or neurodegenerative disease risk [19, 20]. Because HLA-A and HLA-B may also differ in the breadth and character of their peptide-binding repertoires [21, 22], direct measurement of neuronal peptide presentation across donors is likely to be essential for interpreting susceptibility and antigenic targeting in heterogeneous human disease.

Mass spectrometry-based immunopeptidomics provides a direct approach to identifying peptides naturally presented by HLA class I molecules and has been widely applied for antigen discovery [23–25]. Expression of antigen-processing genes or candidate proteins alone does not specify which peptides are processed and displayed on HLA class I molecules, reinforcing the need for direct ligandome measurement [23, 24]. Applying immunopeptidomics to neuronal systems requires scalable neural cultures, approaches to resolve neuron-specific contributions in mixed-lineage preparations, and functional assays that connect peptide discovery to antigen-specific cytotoxicity. While IFNγ-induced upregulation of HLA class I is well established, there is a lack of knowledge on the extent to which human neurons process and present endogenous peptides under inflammatory conditions. In this work, we establish a human iPSC-derived neural aggregate system that enables integrated analysis of neuron-enriched MHC class I immunopeptidomes and functional antigen-specific CD8^+^ T cell engagement in an autologous setting [1].

## Results

### IFNγ stimulation induces upregulation of HLA-A/B/C by human iPSC-derived neural aggregates

**Fig. 1:**
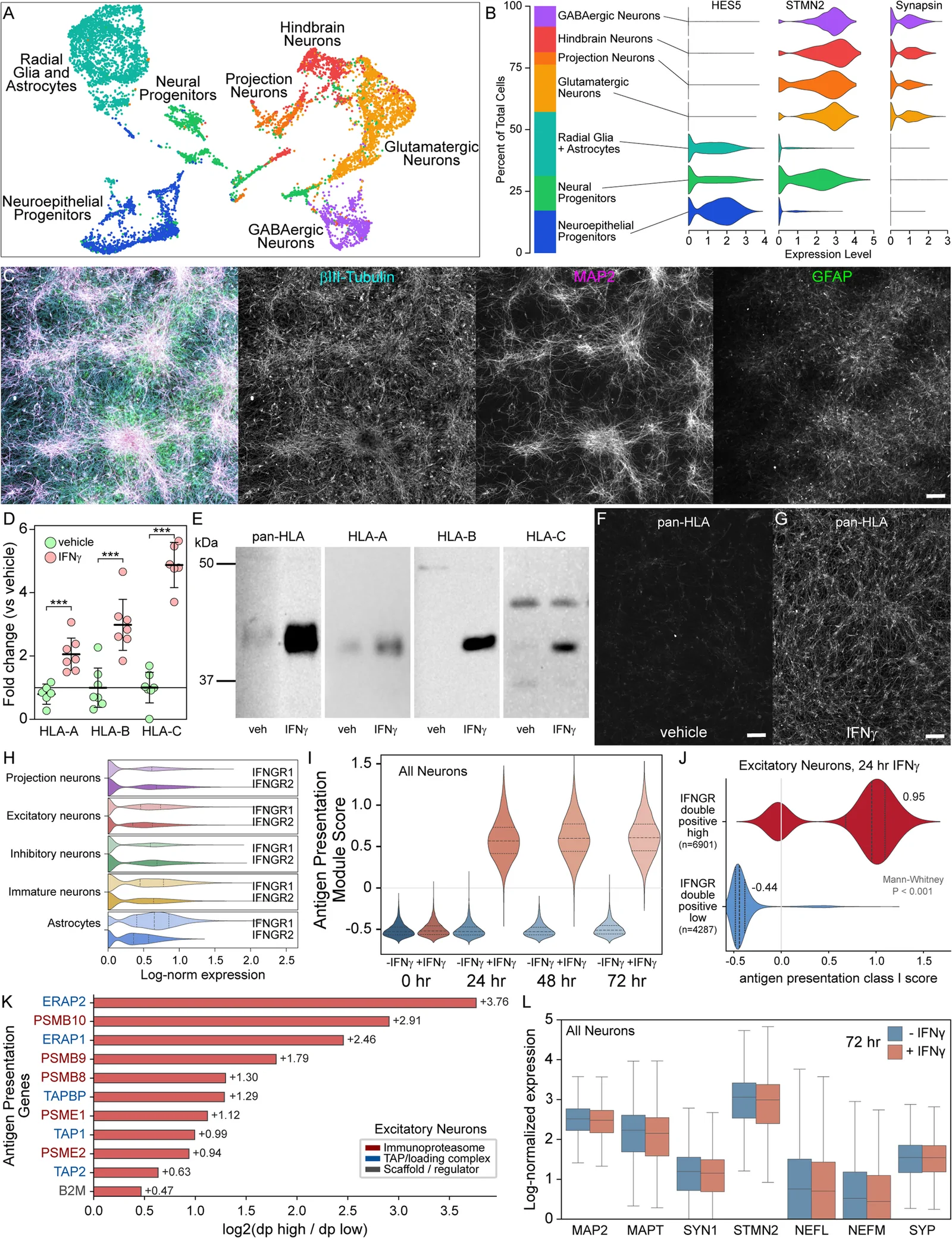
Characterization of the IFNγ-induced HLA class I response in human neural aggregates. **A** UMAP visualization of single-nucleus RNA-seq from HNA cultures, annotated into major neural lineage states including neuroepithelial progenitors, neural progenitors, radial glia/astrocytes, and multiple neuronal subtypes (glutamatergic, projection, hindbrain/spinal, and GABAergic neurons). **B** Cell-type composition (left) and expression distributions (right) for representative markers used to support annotation, including the progenitor marker HES5 and neuronal markers STMN2 and synapsin. **C** Representative immunofluorescence image of HNA cultures showing dense neuronal networks and mixed lineage composition, with single-channel views of βIII-tubulin (neuronal), MAP2 (neuronal dendritic), and GFAP (astrocytic) staining. **D** Quantification of IFNγ-induced HLA class I locus RNA expression, shown as fold-change relative to vehicle across HLA-A, HLA-B, and HLA-C; each symbol represents an independent sample (n = 6 or n = 7) and error bars show mean and 95%CI; ***P < 0.001 by pairwise t-test. **E** Immunoblot analysis of pan-HLA and locus-specific HLA-A, HLA-B, and HLA-C expression in vehicle- and IFNγ-treated HNAs. **F**-**G** Representative pan-HLA immunofluorescence images showing low basal class I staining in vehicle-treated cultures (**F**) and marked upregulation following IFNγ stimulation (**G**). **H** Violin plots showing log-normalized expression of IFNGR1 and IFNGR2 across five consolidated HNA cell type categories in unstimulated cells; neuronal categories shown above, astrocytes below. Internal lines indicate quartiles. **I-L** Single-nucleus RNA sequencing of HNAs treated with IFNγ across a 0–72 h time course (8 conditions: 4 timepoints × ± IFNγ; n = 96,229 cells passing quality control; n = 40,780 neurons across glutamatergic, GABAergic, and other neuronal subtypes pooled for aggregate analysis). **I** Distribution of antigen presentation module scores across all neurons by condition and time. The module aggregates expression of TAP1, TAP2, TAPBP, B2M, PSMB8, PSMB9, PSMB10, PSME1, ERAP1, ERAP2, NLRC5, IRF1, and STAT1. Untreated neurons remain at baseline across all timepoints; IFNγ-treated neurons at 0 h are indistinguishable from untreated, while 24, 48, and 72 h IFNγ-treated neurons show pronounced upward shifts of similar magnitude. Internal lines indicate distribution quartiles. **J** Antigen presentation class I module scores in IFNγ-stimulated excitatory neurons at 24 h, comparing dual-positive-high (IFNGR1+IFNGR2+; n = 6,901) and dual-positive-low (IFNGR1+IFNGR2-, IFNGR1-IFNGR2+, IFNGR1-IFNGR2-; n = 4,287) subclusters. Median values annotated; Mann-Whitney U, *P* < 0.001. **K** Log2 fold-change of individual antigen presentation machinery genes between dual-positive-high and dual-positive-low excitatory neurons at 24 h IFNγ+ (log2[dp-high mean / dp-low mean]). Gene labels colored by functional class: immunoproteasome subunits (PSMB8, PSMB9, PSMB10, PSME1, PSME2), TAP/loading complex and ER aminopeptidases (TAP1, TAP2, TAPBP, ERAP1, ERAP2), and scaffold/light chain (B2M). **L** Expression of canonical pan-neuronal identity markers (MAP2, MAPT, SYN1, STMN2, NEFL, NEFM, SYP) in neurons from untreated (blue) versus IFNγ-treated (orange) conditions, restricted to the 72 h timepoint. Boxes represent the interquartile range, lines indicate medians, and whiskers extend to 1.5× IQR. Outliers are not shown. The overlapping distributions confirm that IFNγ exposure does not alter neuronal identity. Scale bars in (C, F, and G) represent 100 μm

Human neural aggregates (HNAs) were prepared from healthy control and patient-derived induced pluripotent stem cells (iPSCs), as we previously described [15]. Briefly, neural stem cells (NSCs) induced by dual SMAD inhibition of iPSCs were differentiated using db-cAMP, BDNF, IGF1, GDNF, and B27 supplement. Analysis of cellular constituents in the aggregates after 14–21 days of maturation using single nucleus RNAseq indicated enrichment for mature glutamatergic and GABAergic neurons, along with astrocytes, radial glia, and early and late neural progenitors (Fig. 1A and B).

Evaluation of βIII-tubulin, MAP2, and GFAP expression by immunolabeling and confocal fluorescence microscopy confirmed dense neuronal aggregates with extensive neurites situated within clusters of astrocytes (Fig. 1C). Stimulation of HNAs with IFNγ (100 ng/mL) for 72 h induced differential changes in expression of HLA class I genes relative to vehicle-treated HNAs, with a 2-fold increase in HLA-A (*P* = 0.0004 by t-test; Hedges’ g = 2.6), a 3-fold increase in HLA-B (*P* = 0.0005 by t-test; Hedges’ g = 2.4), and a 5-fold increase in HLA-C (*P* < 0.0001 by t-test; Hedges’ g = 6.0) (Fig. 1D). This observation was confirmed by western blotting HNA lysates (Fig. 1E) and by surface labeling for pan-HLA in unstimulated (Fig. 1F) and IFNγ-stimulated HNAs (Fig. 1G). We conclude that cultures of mature human neurons derived from human iPSCs upregulate expression of HLA class I molecules in response to stimulation with IFNγ, with particular enrichment at both the RNA level and protein amount for HLA-B and HLA-C.

To address whether IFNγ acts directly on neurons rather than through paracrine relay from glia, we examined expression of both IFNγ receptor subunits across consolidated cell type categories. Because IFNGR2 is the obligate signaling subunit required to assemble the functional receptor complex [26, 27], cells co-expressing both subunits (dual-positive) represent the population competent for direct IFNγ responsiveness. Both subunits were constitutively expressed at baseline in all five categories, including all mature neuronal populations (Fig. 1H); dual-positive fractions ranged from 22% in projection neurons to 44% in excitatory neurons and 66% in astrocytes, with lower neuronal detection consistent with the reduced transcript abundance of post-mitotic neurons in snRNA-seq.

To define the kinetics and cell-type specificity of HLA class I antigen presentation in human iPSC-derived neurons, we performed 10x Genomics Flex single-nucleus RNA sequencing on human neural aggregates treated with recombinant IFNγ for 0, 24, 48, and 72 h, with matched untreated controls at each timepoint. Across 8 samples processed in two libraries, we recovered 96,229 single nuclei passing quality and doublet filtering. Cells were classified into major lineages by hierarchical scoring of canonical marker gene modules: 40,780 neurons (42.4% of cells), 52,159 progenitor-lineage cells (54.2%), and 3,290 cells assigned to transitional or unassigned categories. The neuronal compartment was further subdivided into glutamatergic, GABAergic, and other neuronal populations on the basis of subtype-specific marker expression. Marker-based assignment was independent of treatment condition: the proportions of each cell type were essentially identical between IFNγ-treated and untreated samples, confirming that IFN-γ does not transdifferentiate cells across major lineage boundaries. We assembled an antigen presentation module comprising 13 canonical genes spanning the major functional steps of class I peptide loading and presentation: peptide generation by the immunoproteasome (PSMB8, PSMB9, PSMB10, PSME1), peptide trimming in the endoplasmic reticulum (ERAP1, ERAP2), peptide transport (TAP1, TAP2), peptide loading (TAPBP), the obligate class I light chain (B2M), and the master transcriptional regulators of the program (NLRC5, IRF1, STAT1). At baseline, neurons expressed essentially none of these genes (median module score − 0.50; Fig. 1I) and the baseline distribution was stable across the entire 72-hour time course in untreated samples. IFNγ-treated neurons at the 0-hour timepoint were indistinguishable from untreated controls (Fig. 1I) confirming that the IFNγ effect is treatment-dependent rather than a sample-handling artifact. By 24 h of IFNγ exposure, the antigen presentation module was strongly induced: median module score rose from − 0.50 to + 0.57, an absolute shift exceeding one standard deviation of the baseline distribution. The response showed essentially no further increase at 48–72 h; median module scores at these later timepoints were + 0.60 and + 0.61, respectively. The cumulative response distribution across all neurons (Supplemental Fig. 3A) confirms this plateau across the entire range of response intensities: the 24-, 48-, and 72-hour IFNγ-treated curves are overlaid, indicating that not only the average response but the full distribution of cellular response magnitudes is established by 24 h and stable thereafter.

Examination of individual module genes confirmed that the response is broad and coordinated across the antigen presentation pathway rather than driven by one or two outlier genes (Supplemental Fig. 3B). At 24 h of IFNγ exposure, neurons induced TAP1 (mean log-normalized expression 0.0 → 1.4), B2M (0.0 → 2.6), STAT1 (0.2 → 2.6), NLRC5 (0.0 → 1.3), IRF1 (0.0 → 1.2), TAP2 (0.0 → 1.1), TAPBP (0.1 → 0.8), PSME1 (0.3 → 1.0), PSMB8 (0.0 → 0.2), and PSMB9 (0.0 → 0.2), with each gene reaching plateau expression by the 24 h timepoint. The single-nucleus data further allowed us to quantify response heterogeneity across the neuronal population. By 24 h of IFNγ exposure, essentially all neurons had engaged the antigen presentation program above baseline (> 99% of neurons exceeded a module score of zero, the upper boundary of the IFNγ-untreated distribution). However, the magnitude of the response varied across cells: approximately 89% of neurons reached a moderate response level (score > 0.3), 45% reached a strong response (score > 0.6), and 10% reached a very strong response (score > 0.9). This pattern was preserved at 48 and 72 h, again indicating that the heterogeneity reflects intrinsic differences among neurons rather than progressive temporal recruitment.

Receptor co-expression predicted IFNγ signaling competence. Dual-positive cells (IFNGR1 + IFNGR2 expressing) were distributed non-randomly within every neuron category (all chi-square *P* < 0.001), with the most dual-positivity observed in excitatory neurons (*n* = 17,163 of 40,566; enrichment ratio 1.28; Supplemental Fig. 3C). Sub-clustering excitatory neurons resolved this enrichment to the dorsal spinal cord (ZIC+, HOX-C/A 4–9+) subtype, which comprised the two most dual-positive-enriched subclusters (enrichment ratios 1.69 and 1.65; Supplemental Fig. 3D, 3E), versus the not-enriched remainder of predominantly VGLUT2 + and LHX1/LBX1 + neurons. Following IFNγ stimulation, dual-positive-high excitatory neurons (pooled across all timepoints) reached a median antigen presentation class I score of + 0.95 versus − 0.44 in dual-positive-low neurons (Mann-Whitney *P* < 0.001; *n* = 6,901 vs. 4,287; Fig. 1J). Per-gene comparison of dual-positive-high against dual-positive-low excitatory neurons in response to 24 h IFNγ stimulation (Fig. 1K; *n* = 1,758 dual-positive-high vs. 2,771 dual-positive-low) showed the differential was largest for the inducible immunoproteasome subunits and ER aminopeptidases (ERAP2 log2FC = 3.76, PSMB10 = 2.91, ERAP1 = 2.46, PSMB9 = 1.79, PSMB8 = 1.30) and modest for the constitutively-expressed class I light chain B2M (0.47). Together these data indicate that both receptor subunits are constitutively expressed in HNA neurons and that single-cell receptor co-expression predicts the magnitude of antigen presentation induction, consistent with direct cell-autonomous IFNγ signaling.

Finally, these transcriptional responses did not come at the cost of neuronal identity or dedifferentiation. Canonical pan-neuronal markers including MAP2, MAPT, SYN1, STMN2, NEFL, NEFM, and SYP showed essentially overlapping expression distributions in IFNγ-treated and untreated neurons (all subclusters) across the 24–72 h treatment window (Fig. 1L). Cells that engaged the antigen presentation program retained the molecular signature of mature neurons throughout the treatment period. Together, these data establish that human iPSC-derived neurons in our system rapidly acquire the transcriptional machinery required for HLA class I antigen presentation in response to IFNγ. The response engages canonical regulators (NLRC5, STAT1, IRF1) and downstream effectors of peptide processing, transport, and loading, reaches its plateau within 24 h, is sustained through 72 h, and does not perturb neuronal identity.

### Neuron-enriched exogenous antigens are presented on HLA class I

**Fig. 2:**
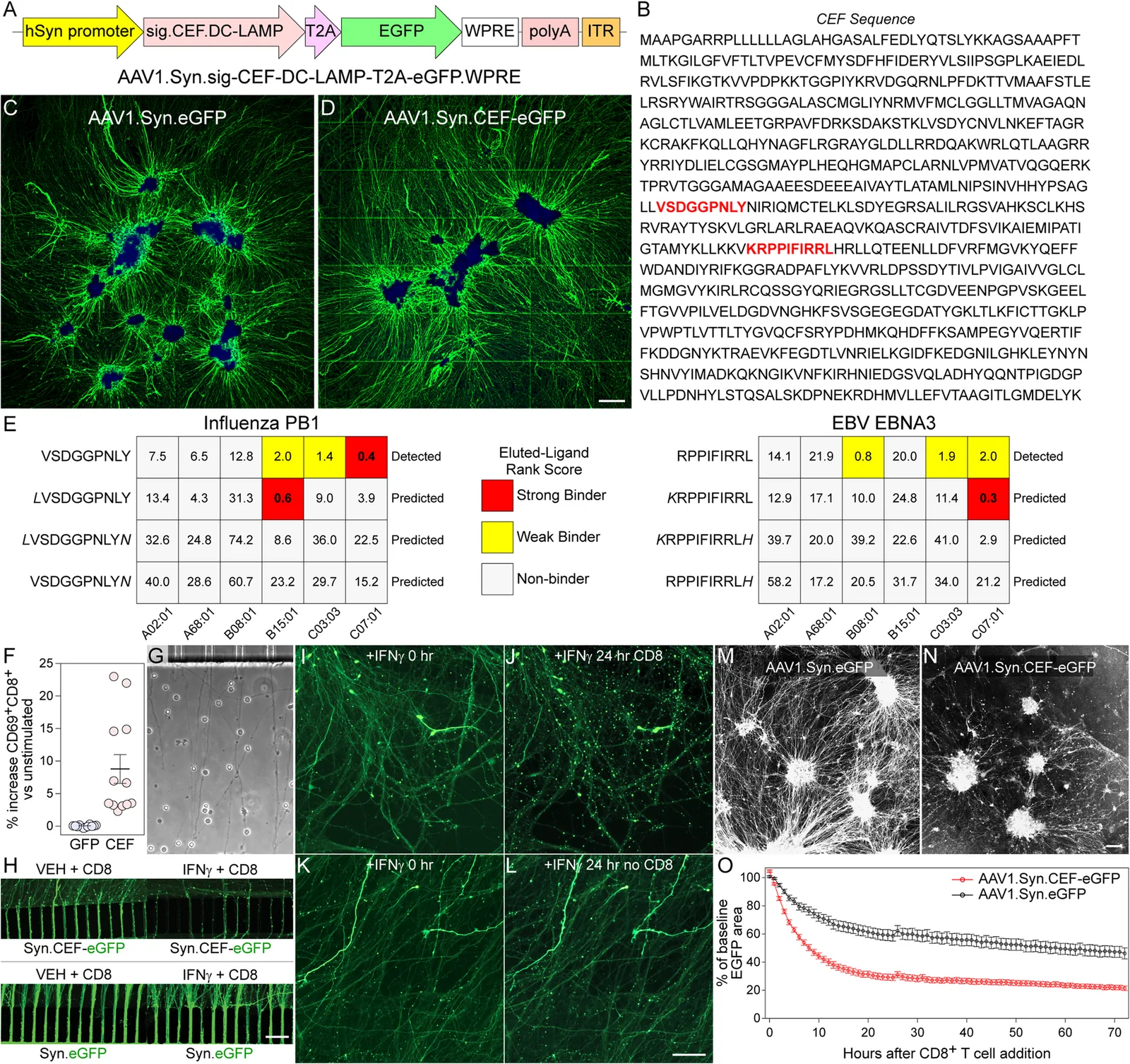
Neuron-restricted expression of defined exogenous antigens drives antigen-specific CD8⁺ T cell recognition and consequent neural injury. **A** Schematic of the AAV expression cassette used to drive neuron-specific expression of a polyepitope antigen (CEF = CMV, EBV, influenza) under the human synapsin promoter (hSyn), coupled to eGFP via a self-cleaving T2A sequence (AAV1.Syn.sig-CEF-DC-LAMP-T2A-eGFP.WPRE). **B** Amino-acid sequence of the expressed antigen cassette with the embedded viral epitopes identified below (influenza PB1 and EBV EBNA3) shown in red. **C** Representative fluorescence images of control- (AAV1.Syn.eGFP) and (**D**) CEF-transduced (AAV1.Syn.CEF-eGFP) neural cultures demonstrating robust neuron-restricted eGFP expression and neurite-rich morphology. **E** NetMHC-based eluted-ligand rank predictions for CEF epitopes (influenza PB1 and EBV EBNA3) identified by LC-MS/MS analysis of peptides bound to neural cell HLA class I following IFNγ stimulation; heatmaps summarize predicted binding class (strong/weak/non-binder) across donor HLA class I allotypes and detected peptides are indicated alongside predicted single amino acid extension variants consistent with antigen processing. **F** Quantification of antigen-specific CD8⁺ T cell activation following co-culture with control- (AAV1.Syn.eGFP) or AAV1.Syn.CEF-eGFP transduced neurons for 72 h, shown as the percent increase in CD69⁺CD8⁺ T cells percentage relative to unstimulated controls; symbols indicate individual replicates and bars summarize mean and 95%CI. **G** Representative brightfield image demonstrating T cell interaction with neurites in the microfluidically isolated device co-culture condition. **H** Representative images showing antigen- and IFNγ-dependent neurite injury in AAV1.Syn.eGFP or AAV1.Syn.CEF-eGFP expressing neurons following addition of antigen-specific CD8⁺ T cells into the axon chamber of the microfluidic device. **I-L** Time- and condition-specific fluorescence images showing loss of neurite integrity only in the presence of IFNγ and antigen-specific CD8⁺ T cells. **M-N** Representative images of profound neurite loss following addition of antigen-specific CD8+ T cells into the cell body chamber only in the AAV1.Syn.CEF-eGFP transduced cultures (**N**). **O** Longitudinal live-cell imaging quantification of eGFP+ neurite loss, expressed as percent of baseline eGFP area over time following CD8⁺ T cell addition, comparing AAV1.Syn.CEF-eGFP expressing (red) to control AAV-transduced (black) neurons. Scale bar in (D) is 250 μm and refers to (C-D). Scale bar in (H) is 100 μm; scale bar in (L) is 100 μm and refers to (I-L); scale bar in (N) is 100 μm and refers to (M-N)

To determine whether IFNγ-induced HLA class I expression enables neurons to present a defined antigen, we engineered an AAV cassette in which a polyepitope of immunodominant cytomegalovirus, Epstein-Barr virus, and influenza (CEF) epitopes was placed under the neuron-specific synapsin (Syn) promoter, restricting antigen expression to neurons. Inoculation of HNAs with this AAV (AAV1.Syn.sig-CEF-DC-LAMP-T2A-eGFP.WPRE; hereafter designated AAV1.Syn.CEF-eGFP) (Fig. 2A) or a control vector encoding eGFP downstream of the synapsin promoter without CEF (AAV1.Syn.eGFP) resulted in neuron-restricted expression of eGFP (Fig. 2C and D). Exploiting this neuron-specific expression of exogenous antigens, we scaled up production of NSCs and differentiated HNAs to obtain > 1.2 × 10^8^ neural cells, stimulated with IFNγ for 72 h, and processed for immunoprecipitation of HLA class I: peptide complexes followed by LC-MS/MS-based analysis of HLA-bound peptides. This approach yielded 4364 peptides (restricted to 7–25 amino acids in length) from 2411 unique proteins. Notably, two CEF-derived 9-mer peptides were identified: VSDGGPNLY (CEF_374−382_) from the PB1 polymerase of influenza A and RPPIFIRRL (CEF_479−487_) from EBNA3 (Fig. 2B). We analyzed these two peptides in NetMHCpan 4.2 for allele-specific binding [28] and defined eluted-ligand (EL) ranks < 0.5 as “strong binders” and 0.5 < EL < 2.0 as “weak binders” [29]. Across the HLA class I haplotype of the healthy donor used for this experiment (HLA-A02:01, HLA-A68:01, HLA-B08:01, HLA-B15:01, HLA-C03:03, HLA-C07:01), the influenza peptide is predicted to bind strongly to HLA-C07:01 and weakly to HLA-C03:03 and HLA-B15:01 (Fig. 2E). The EBV peptide is predicted to bind weakly to HLA-B08:01, HLA-C03:03, and HLA-C07:01 (Fig. 2E). Because differentially trimmed versions of antigenic peptides may exist, we also analyzed binding of ± 1 terminal amino acid variants of the two detected peptides. This revealed that the N_term_+1 peptide KRPPIFIRRL from EBV (CEF_478−487_) is a strong binder to HLA-C07:01 and the N_term_+1 peptide LVSDGGPNLY from influenza (CEF_373−382_) is a strong binder to HLA-B15:01 (Fig. 2E). We conclude that human iPSC-derived neurons are capable of presenting exogenous antigens on HLA class I following IFNγ stimulation.

### Neuron-enriched exogenous antigens presented on HLA class I elicit CD8^+^ T cell-mediated injury

Given the broad immunity against EBV and influenza, we sought to determine whether CD8^+^ T cells from the autologous iPSC donor used to generate HNAs would recognize the peptides presented on HLA class I and injure the neurons. We transferred AAV1.Syn.CEF-eGFP or AAV1.Syn.eGFP transduced HNAs into a multicompartment microfluidic device that separates neural cell bodies from distal axons, as previously described [15]. In parallel, we expanded T cells from the autologous donor by culturing peripheral blood mononuclear cells (PBMCs) in IL7 for 3 days and we generated dendritic cells (DCs) by culturing PBMCs in GMCSF+IL4 for the same period. Subsequently, DCs were incubated with a CEF peptide pool and co-cultured with the T cells for a further 3 days to drive expansion of CEF-specific T cells. During the same period the HNAs were stimulated with IFNγ (100 ng/mL) to drive HLA class I upregulation. CD8^+^ T cell activation by the CEF-presenting DCs was verified by flow cytometry (Fig. 2F). CD8^+^ T cells were then isolated by negative magnetic selection and added to the axonal compartment of microfluidic devices containing autologous HNAs transduced with AAV1.Syn.CEF-eGFP or AAV1.Syn.eGFP (Fig. 2G). eGFP^+^ axons were imaged repeatedly in the distal chamber, with evidence of extensive injury only in the IFNγ-stimulated cultures incubated with activated CD8^+^ T cells (Fig. 2H-L). Critically, axon injury was strictly antigen-dependent: CD8^+^ T cells only substantially injured axons in HNAs transduced with AAV1.Syn.CEF-eGFP and only in such HNAs treated with IFNγ (Fig. 2H and J). HNAs only transduced with AAV1.Syn.eGFP, even when treated with IFNγ, were resistant to CD8^+^ T cell-mediated injury (Fig. 2H). Introduction of anti-CEF CD8^+^ T cells into the cell body chamber elicited even more robust axonal injury, but only in HNAs transduced with AAV1.Syn.CEF-eGFP (Fig. 2M and N). Serial live-cell imaging demonstrated progressive loss of neurites starting shortly after the addition of CD8^+^ T cells in the AAV1.Syn.CEF-eGFP transduced HNAs but not in the AAV1.Syn.eGFP transduced cells (Fig. 2O). We conclude that human iPSC-derived neurons are competent to present exogenous antigens on HLA class I following IFNγ stimulation, resulting in axon injury by antigen-specific CD8^+^ T cells.

### Neuron-enriched candidate antigens are presented on HLA class I

**Fig. 3:**
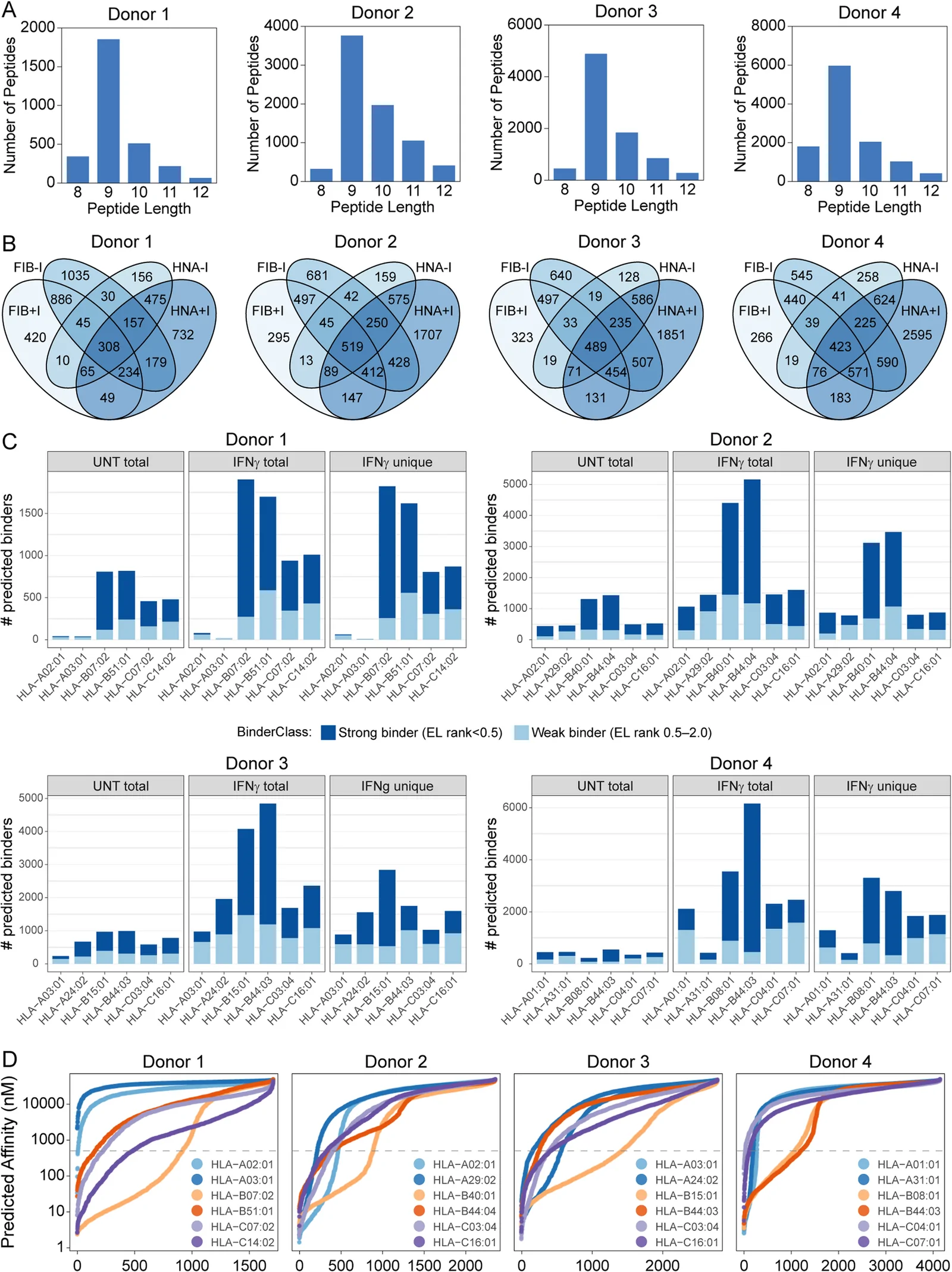
Multi-donor mapping of the IFNγ-induced neural immunopeptidome shows relative enrichment of predicted HLA-B-associated peptides among predicted binders. **A** Length distribution of HLA class I-eluted peptides detected by LC-MS/MS from IFNγ-stimulated neural cultures across four donors, showing enrichment for 8-12-mers with a predominance of 9-mers. **B** Overlap analysis of candidate antigenic protein repertoires across conditions within each donor, highlighting the subset of peptides unique to IFNγ-stimulated neural cultures relative to matched controls. FIB-I = unstimulated fibroblasts; FIB + I = IFNγ-stimulated fibroblasts; HNA-I = unstimulated HNAs; HNA + I = IFNγ-stimulated HNAs. **C** NetMHC-based binding classifications for donor-matched HLA class I allotypes, shown for untreated (UNT) total, IFNγ total, and IFNγ-unique peptide sets; stacked bars indicate predicted strong (dark blue) versus weak (light blue) binders by eluted-ligand rank thresholds, illustrating a consistent enrichment of predicted binders on HLA-B allotypes across donors. **D** Rank-ordered predicted affinity plots for IFNγ-unique 9-mers, stratified by donor HLA allotype; each curve represents peptides assigned to the best-predicted allotype, visualizing the high-affinity tail and the enrichment of specific HLA-B allotypes in multiple donors; x-axis shows cumulative rank index of analyzed peptides; dashed line shows 500 nM cutoff

Using the same HLA immunoprecipitation assay and LC-MS/MS we characterized the immunopeptidome in unstimulated and IFNγ-stimulated HNAs and fibroblasts from 4 different donors. Donor fibroblasts and iPSC-derived NSCs differentiated into HNAs were expanded to > 1.2 × 10^8^ cells, stimulated with IFNγ or vehicle for 72 h, and processed for immunoprecipitation of HLA class I: peptide complexes, as above. As expected, the majority of peptides isolated from the HNAs were between 8 and 12 amino acids in length, with a predominance of 9-mers (Fig. 3A), so the subsequent downstream analysis focused primarily on peptides within this size range. To assess reproducibility of peptide recovery, replicate iPSC-derived NSC cultures generated from the same donor were independently analyzed by IP-LC-MS/MS at two separate proteomic core facilities (University of Arizona and Mayo Clinic Rochester), demonstrating substantial overlap in recovered peptides/proteins and consistent HLA-binding motifs across datasets (Figure S1). Within each subject we compared conditions to identify peptides (8–12 residues) that were unique to the IFNγ-stimulated HNAs relative to IFNγ-stimulated fibroblasts and unstimulated HNAs and fibroblasts. Of 3016 total peptides in Donor 1 HNAs stimulated with IFNγ, 1904 were unique to the IFNγ-stimulated neurons and not detected in the unstimulated HNAs or the fibroblasts. Donor 2 had 5534 unique out of 7564 total; Donor 3 had 5953 unique out of 8324 total; and Donor 4 had 9556 unique out of 11,305 total peptides. From a total of 2199 protein hits, Donor 1 had 732 that were unique to the IFNγ-stimulated HNAs. Of 4127 total proteins, Donor 2 had 1707 unique; of 4324 total proteins, Donor 3 had 1851 unique; and of 5287 total proteins, Donor 4 had 3939 unique hits (Fig. 3B).

NetMHCpan 4.2 was used to predict the affinity of each neural cell-derived peptide for the corresponding HLA class I allotypes present in each subject (Fig. 3C). This analysis revealed a relative enrichment of HLA-B-binding peptides, outnumbering HLA-A and HLA-C binding peptides, both in the total immunopeptidome and in the peptidome unique to IFNγ-stimulated HNAs. Identification of strong binders (top 0.5%) and weak binders (top 2%) likewise indicated a preponderance of strong binding peptides on HLA-B for all 4 subjects. Analysis of the predicted affinity for the unique IFNγ-stimulated 9-mer peptidome for each subject using NetMHCstabpan 1.0 revealed that a single HLA-B allotype accounted for the majority of strong binding peptides in 3 of 4 donors (Donor 1, 3, 4), while the fourth donor showed strong binding to both HLA-B allotypes (Fig. 3D).

**Fig. 4:**
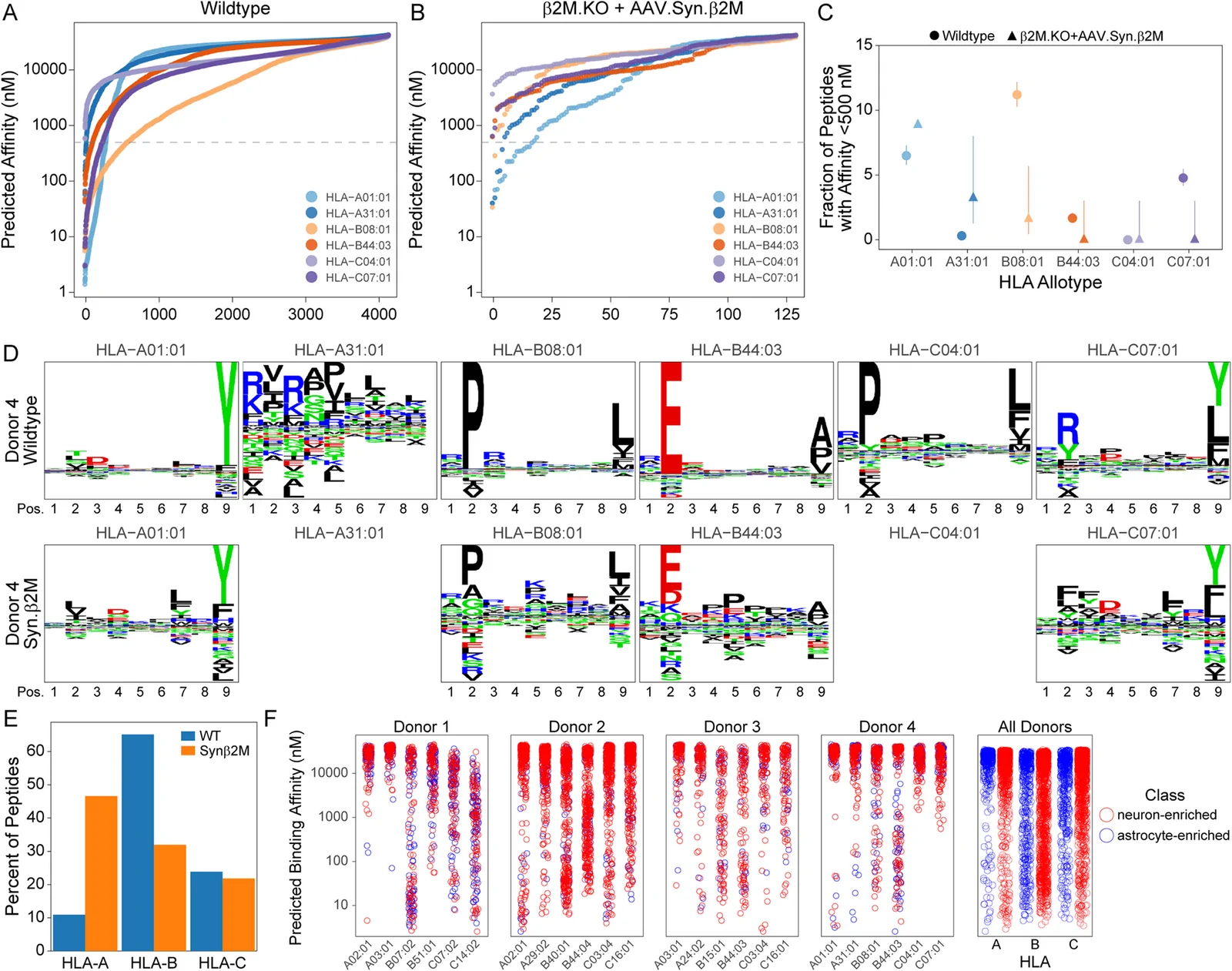
Neuron-restricted β2M rescue alters locus weighting and nominates several recurring and unique neuron-enriched candidate antigens. **A-B** Rank-ordered predicted affinity plots for Donor 4 WT (**A**) versus the β2M knockout line from the same donor rescued with neuron-restricted β2M expression (β2M.KO + AAV.Syn.β2M) (**B**), showing the distribution of predicted affinities for HLA class I bound peptides across HLA class I allotypes; x-axis shows cumulative rank index of analyzed peptides; dashed line shows 500 nM cutoff. **C** Fraction of peptides below an affinity threshold (< 500 nM) predicted to bind to each HLA allotype in WT versus the neuron-restricted β2M rescue, highlighting a shift in the high-affinity fraction across loci/allotypes. **D** Motif deconvolution of 9-mer repertoires shows inferred allele-restricted anchor preferences for each allotype between Donor 4 WT (top) and the neuron-restricted β2M rescue (bottom). **E** Summary of inferred locus composition (percent of peptides assigned to HLA-A, HLA-B, or HLA-C) comparing WT and neuron-restricted β2M rescue. **F** Predicted affinity scatter plots across donor-specific HLA allotypes for peptides derived from proteins classified by single-nucleus RNA-seq-informed enrichment (“neuron-enriched” versus “astrocyte-enriched”), shown for each donor and pooled across donors

Using CRISPR/Cas9 editing, β2-microglobulin (β2m) was deleted from Donor 4 NSCs. According to inference of CRISPR edits (ICE) in the sequencing data from these cells we achieved approximately 98% knockout of β2M. HLA immunoprecipitation and LC-MS/MS analysis of Donor4_β2M.KO HNAs identified only 34 total peptides, of which 16 were 8-12-mers and 5 were 9-mers (compared to 6995 total, 6703 8-12-mers, and 4189 9-mers in the matched IP-MS run for the wildtype Donor 4 cells in this specific experiment). These peptides were likely derived from low-level contaminant carryover or non-specific binding. To refine the neuron-enriched immunopeptidome within this framework, we reintroduced the β2M protein in the Donor 4 β2M.KO HNAs by transducing with AAV1-Syn1.hB2M/T2A/eGFP: oPRE (referred to as AAV1.Syn.β2M). This led to the identification of 602 total peptides, 366 8-12-mers, and 141 9-mers derived from neurons. Of the 8-12-mers, 131 peptides were predicted as strong binders and 182 as weak binders using eluted-ligand ranking. Comparing the predicted peptide binding affinity for the wildtype Donor 4 HNAs and the AAV1.Syn.β2M reconstituted β2M.KO HNAs (Synβ2M) showed a relative enrichment of predicted HLA-B*08:01 binders within the high-affinity pool in the wildtype (Fig. 4A) while HLA-A*01:01 had the strongest representation in the reconstituted cells (Fig. 4B). Because the reconstituted condition had ~ 30-fold fewer total peptides scored for binding relative to the wildtype (141 vs. 4189 9-mers used in the analysis), the distribution is likely undersampled, potentially skewing the HLA locus representation and missing rare high-affinity events. To facilitate a direct comparison between the wildtype and the reconstituted neural cell immunopeptidomes, we recast the by-allotype data as fraction of peptides with affinity < 500 nM relative to the total number of peptides identified (Fig. 4C). This normalization strategy revealed that the pattern of relative HLA-B enrichment in the wildtype was largely absent in the β2M reconstituted neurons and the high-affinity fraction was HLA-A weighted. To test whether the apparent loss of HLA-B predominance in Synβ2M could be explained by undersampling, we performed a depth-matched resampling analysis. Specifically, we repeatedly drew random sets of 141 WT 9-mer peptides (matching the Synβ2M 9-mer count), reassigned each peptide to its best-predicted HLA allotype (lowest predicted affinity), and quantified the resulting HLA-A/B/C distribution across 5000 iterations. Across all resamples, WT showed relative enrichment of predicted HLA-B-associated peptides and did not converge toward the Synβ2M locus distribution. While this suggests that the observed WT vs. Synβ2M difference is unlikely to be an artifact of sampling depth, the approach does not exclude biological differences in antigen processing and loading or in peptide source pools between the WT condition and the neuron-restricted β2M rescue. Given the cellular heterogeneity of the HNAs, the identified peptide repertoire likely reflects contributions from multiple neuronal and non-neuronal cell populations. The primary purpose of this experiment was to confirm the HLA class I dependency of the detected peptide repertoire; the altered peptide composition observed upon neuron-restricted reconstitution likely reflects non-physiological expression constraints and should be interpreted with caution. While neuron-restricted β2M supports a neuronal contribution, the reduced peptide yield in this condition suggests that a subset of peptides identified in bulk analyses may originate from non-neuronal cells.

To assess whether WT and Synβ2M peptidomes differed in allele-specific anchor preferences, consistent with differential HLA allotype binding, we deconvoluted the 9-mer repertoires using MHCMotifDecon 1.2 to infer likely HLA class I restrictions and to visualize anchor motifs (Fig. 4D). We additionally summarized the inferred restriction fractions by locus (HLA-A vs. HLA-B/C) and compared these estimates to NetMHC-based best-allele assignments (Fig. 4E). To further test the hypothesis that neuron-restricted peptides exhibit an HLA-A preference relative to glial cell-derived peptides, we used our single-nucleus RNAseq data to assign a cell source classification (“neuron-enriched” vs. “astrocyte-enriched”) to each peptide source protein identified by immunopeptidomics analysis of the four main donors. We then plotted the predicted affinity for each donor-specific HLA allotype (Fig. 4F). Consistent with the IFNγ-unique immunopeptidome bias toward neuron-associated proteins, the affinity plots were dominated by neuron-enriched calls. However, across all 4 donors, we did not observe enrichment of HLA-A assignment for peptides derived from neuron-enriched proteins relative to astrocyte-enriched proteins (OR = 1.08, Fisher’s *P* = 0.51), and high-affinity peptides from either cell classification were most frequently assigned to HLA-B allotypes (Fig. 4F). Together with donor-to-donor variability in HLA-A representation for high-affinity assignments and the depth mismatch observed in the Synβ2M dataset, these results suggest that the WT neural immunopeptidome is relatively HLA-B skewed, while the apparent HLA-A-weighting in the Synβ2M condition is suggestive but should be interpreted cautiously (Figs. 3 and 4).

**Fig. 5:**
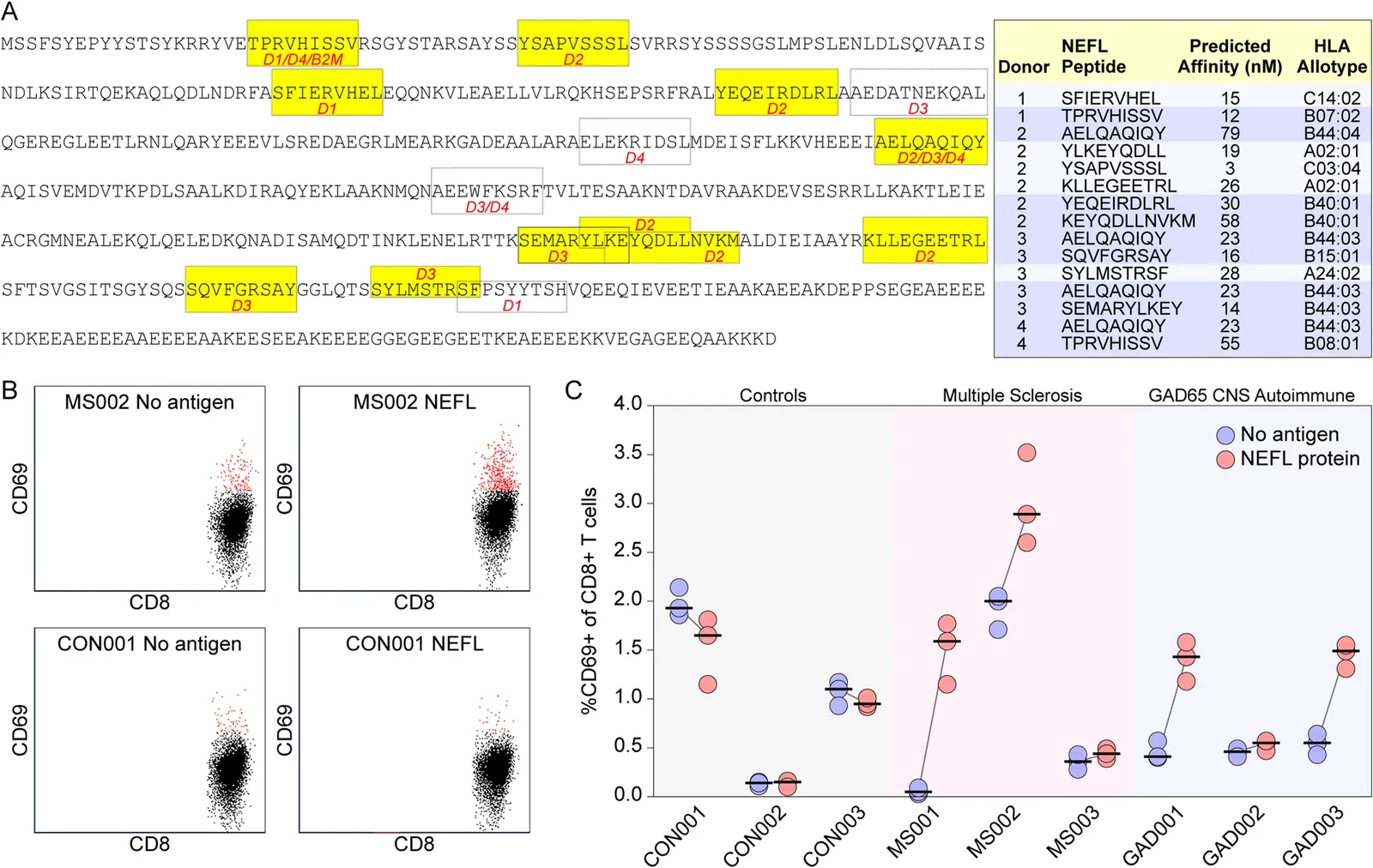
CD8+ T cell responses to NEFL whole protein in subjects with autoimmune CNS disease and healthy controls. **A** Example of a neuron-restricted antigen, neurofilament light chain (NEFL), recurringly identified in all four donors and presented in the neuron-specific β2M rescue condition; schematic protein map with detected peptides and donor sharing indicated; table inset summarizes representative NEFL-derived peptides with predicted affinity and best-assigned restricting HLA allotype. **B** Representative CD8 vs. CD69 bivariate flow cytometry plots for one representative MS subject (MS002) and one representative healthy control (CON001) following 72 h coculture with autologous antigen-presenting cells pulsed with either no antigen (“No antigen”) or recombinant NEFL whole protein (“NEFL”). Events are gated on live, single, CD3+CD8+ lymphocytes. CD69+ events are shown in red; CD69− events in black. Expansion of the CD69+ population in the NEFL condition relative to the no-antigen condition is visible in MS002 but not in CON001. **C** Quantification of %CD69+ within the CD8+ T cell compartment across the cohort (*n* = 3 healthy controls, *n* = 3 multiple sclerosis, *n* = 3 GAD65 CNS autoimmune; technical triplicates per condition shown as individual values; red = NEFL; blue = no antigen; horizontal bars indicate the per-subject median). Within-subject medians are connected by a thin line linking the no-antigen and NEFL conditions, such that the slope of the connector reflects the magnitude of the antigen-driven response. Background shading distinguishes the three diagnostic groups

Finally, despite donor-to-donor variability at the peptide level, interpretation of exact peptide overlap across donors is complicated by both distinct HLA haplotypes (Supplemental Data) and the incomplete sampling inherent to deep immunopeptidomics workflows. Direct comparison of the IFN-induced immunopeptidomes identified no peptides shared across all four donors, although 156 peptides were shared across three donors and 963 peptides were shared across two donors. Notably, 154 of the 156 peptides shared across three donors were derived from the three subjects carrying HLA-B44 alleles and exhibited a shared GibbsCluster motif consistent with this restriction, supporting the strong influence of HLA haplotype on peptide recovery. At the source protein level, 45 proteins were represented across all four donors. Accordingly, we focused primarily on recurrent source proteins and broader immunopeptidomic patterns across donors rather than strict peptide identity overlap. During the allotype usage analysis we identified 51 neural proteins with peptides represented in the IFNγ-induced immunopeptidome of at least 2 of the 4 donors, with 6 present in three donors and 4 present in all four donors. None of the proteins were observed in the vehicle-treated immunopeptidomes under our filtering criteria. These neural proteins included synaptic and scaffolding factors (APP, CACNB4, CADM1, DTNBP1, GABRQ, GRIA4, KALRN, KIDINS220, MAP1A, MAP2, NLGN2, NETO2, NPTX2, SPTAN1, STON1, SYNE1, TAGLN3), axonal factors (KIF1A, MAP1B, NEFL, KCNQ2, RUFY3, AGTPBP1, INA, TUBB3), and guidance molecules (EPHB3, PLXNA3, PLXNA4, PLXNB2, DOCK7). The microtubule-associated kinase doublecortin-like kinase 1 (DCLK1), for example, was found in the peptidomes from all 4 donors, with predicted high-affinity binding to HLA-A*01:01 (6.3 nM), HLA-B*15:01 (29 nM), and HLA-C*03:04 (12 nM), amongst others. The neuron-restricted neurofilament light protein (NEFL) was highly represented in the immunopeptidomes of all 4 donors (Fig. 5A), with predicted high-affinity binding on multiple HLA allotypes, including presentation on HLA-B molecules in all of the donors. Of particular note, NEFL was also identified in the immunopeptidome of the AAV1.Syn.β2M reconstituted β2M.KO cells, with the peptide TPRVHISSV being found also in Donor 1. Due to the scale requirements of the immunopeptidomics workflow, deep-coverage biological datasets were not generated for all experimental conditions, although cross-laboratory replicate analyses demonstrated reproducible peptide recovery and conserved HLA-binding motifs (Figure S1). CD8 + T cell responses to NEFL whole protein were assessed by AIM assay in healthy controls (*n* = 3), multiple sclerosis (*n* = 3), and GAD65-associated CNS autoimmunity (*n* = 3) (Fig. 5B and C). All subjects mounted measurable responses to the CEF peptide positive control (background-corrected median 4.27%, range 1.12–14.30%; not shown), confirming that CD8 + T cells could respond to stimulation across the cohort. By contrast, NEFL-driven CD8 activation was heterogeneous: four of six subjects with autoimmune CNS disease (two MS, two GAD65) responded to NEFL, whereas no healthy control did. Across the full cohort, background-corrected NEFL response was higher in autoimmune subjects than in controls, with complete rank separation between groups (Mann-Whitney U = 18, two-sided *P* = 0.024). Given the small control sample (*n* = 3), this observation is preliminary and supports the existence of NEFL-reactive CD8 + T cells in a subset of patients with autoimmune CNS disease rather than establishing a robust group-level difference.

We conclude that IFNγ drives presentation of a broad set of candidate neural antigens across donors and that WT HNA immunopeptidomes show relative enrichment toward HLA-B-associated peptides in our system. In one donor, neuron-restricted rescue of β2M protein shifts the high-affinity fraction toward HLA-A presentation, supporting the possibility that neuronal restriction can alter allotype usage, although additional depth-matched experiments will be required to fully separate sampling artifacts from biology. Together, these findings identify neurofilament light chain as a recurrent neuron-derived antigen within the IFNγ-induced immunopeptidome that is capable of eliciting antigen-responsive T cell activation in multiple sclerosis patient-derived cultures.

## Discussion

In this study we established an experimental framework for the analysis of neuron-enriched HLA class I antigen presentation, the discovery of subject-specific neuroimmunopeptidomes, and the assessment of antigen-specific anti-neuronal CD8^+^ T cell-mediated injury. The primary advance of this study is the integration of human neuronal immunopeptidomics with autologous antigen-specific functional assays in a scalable iPSC-derived platform. We showed that human iPSC-derived neural stem cell (NSC)-derived human neural aggregates upregulate HLA class I expression in response to IFNγ stimulation (Fig. 1) and that neurons in these aggregates become competent to present peptides derived from exogenous antigens on class I following IFNγ stimulation (Fig. 2). We also observed that this presentation has functional consequences, and that iPSC-derived neurons presenting exogenous antigen are targeted by antigen-specific autologous CD8^+^ T cells, resulting in injury and neurite loss (Fig. 2). Using the same HLA immunoprecipitation approach, we characterized the immunopeptidome and predicted HLA allotype binding interaction in HNAs from 4 different donors, revealing a relative enrichment of HLA-B-mediated presentation of high-affinity peptides across all subjects (Fig. 3). We used neuron-restricted β2-microglobulin reconstitution to enrich for neuron-associated peptide presentation and characterize the IFNγ-stimulated neuroimmunopeptidome (Fig. 4). Finally, we found that neurofilament light chain-derived peptides are ubiquitously presented on neuron-enriched HLA class I across donors and across multiple HLA allotypes, and that NEFL protein evokes activation of antigen-responsive CD8^+^ T cells derived from autoimmune patients (Fig. 5).

These findings extend our previous observation that IFNγ drives retrograde induction of HLA class I molecules and the antigen presentation machinery in human neuron-enriched aggregates [15] and that CD8^+^ T cells recognize neuron-restricted antigens in a mouse model of demyelination [16]. We characterized a large neural HLA class I ligandome that was comprised of canonical 8-12-mers and enriched for 9-mers, as expected for a class I immunopeptidome. Independent cross-laboratory analysis of replicate neural cultures further demonstrated reproducible recovery of peptide repertoires and conserved HLA-binding motifs across datasets (Figure S1). Although longer peptides were detected within the recovered ligandome, interpretation of non-canonical peptide lengths should be approached cautiously, as extended HLA class I ligands may adopt atypical binding conformations and are less reliably evaluated by our prediction algorithms. We observed a robust IFNγ-induced repertoire that was unique from both the basal, unstimulated repertoire and from the immunopeptidome of autologous fibroblasts. These data are consistent with a model in which local neuroinflammation, as might be found in demyelinating lesions, around proteinopathic aggregates, and near other degenerative sites within the CNS [30] leads to not only upregulation of neural HLA class I molecules and the antigen loading machinery, but also to a remodeling of the peptide repertoire shaped by immunoproteasome activation [15, 16].

Our single-nucleus transcriptomic analysis demonstrates that human iPSC-derived neurons mount a rapid, coordinated, and sustained antigen presentation response to IFNγ. The kinetics finding has both biological and practical significance. The 24-hour plateau indicates that the transcriptional commitment to class I antigen presentation engages near-maximally within the first day of cytokine exposure, consistent with the directly inducible nature of NLRC5 and the canonical IFNγ-JAK1/2-STAT1 signaling axis. We note one important technical consideration: the 10x Flex Human probe panel used in this experiment does not include classical HLA class I heavy chain loci (HLA-A, HLA-B, HLA-C) owing to the substantial allelic polymorphism at these loci that renders reference-based probe design unreliable. Our single-nucleus analysis therefore measures the IFN-γ-induced transcriptional machinery required for class I assembly and surface presentation but does not directly capture induction of the heavy chain genes themselves.

To directly address whether neurons within HNAs are capable of responding autonomously to IFNγ, we examined constitutive expression of the IFNγ receptor subunits IFNGR1 and IFNGR2 in unstimulated cells across consolidated cell type categories (Fig. 1H). Both subunits were detectable across all five populations, including all mature neuronal categories. The fraction of cells co-expressing both subunits, and therefore the population competent to assemble a functional signaling-capable receptor complex, ranged from 22% in projection neurons to 44% in excitatory neurons and 66% in astrocytes. Lower co-expression rates in mature neurons relative to astrocytes are consistent with the lower transcript abundance characteristic of post-mitotic neurons in single-nucleus preparations and do not necessarily reflect receptor absence. These observations are consistent with prior reports of constitutive IFNγ receptor expression and direct IFNγ responsiveness in neurons, including iPSC-derived systems [31, 32].

A prominent feature of our current findings was the relative bias toward HLA-B presentation across multiple donors with very different HLA haplotypes. Prediction-based allele assignment indicated that HLA-B-binding peptides outnumbered HLA-A- and HLA-C-binders in the total repertoire and particularly in the high-affinity subset (Fig. 3). Locus-specific promoter differences in interferon-mediated induction of HLA class I genes [18] may explain HLA-B enrichment within the context of IFNγ-mediated inflammation, though it is notable that our neuron-restricted β2-microglobulin reconstitution experiment suggested that HLA-A may also be relevant to neural peptide presentation. It is possible that these disparate findings are associated with differential affinity thresholds for peptide binding, with HLA-B representing a more curated, high-affinity repertoire relative to HLA-A. Our observations support this model, with analysis of binding motifs in the wildtype Donor 4 cells and the synapsin-specific β2-microglobulin reconstituted Donor 4 β2M.KO cells showing preservation of the heavy-chain binding pocket selectivity but reduced binding stringency on HLA-B allotypes (marked by broader residue noise) in the neuron-enriched results (Fig. 4D).

The present work focused specifically on IFNγ because of its canonical role in neuronal HLA-I induction; however, future studies should determine how IFNβ, TNFα, and other inflammatory mediators reshape neuronal immunopeptidomes. In the pancreas, interferon signaling (IFNα) shifts the β-cell immunopeptidome from a resting HLA-A predominance to an HLA-B-restricted repertoire and patients with type 1 diabetes exhibit preferential HLA-B hyperexpression relative to non-diabetics. Moreover, islet-infiltrating CD8^+^ T cells recognize granule peptides that are restricted to HLA-B [33]. However, HLA-B allotypes exhibit differential dependence on the canonical peptide-loading machinery, resulting in a broader and more diverse peptide pool under stress or inflammatory conditions [34, 35]. In a CNS context where interferon signaling and proteostasis changes may reshape the processing and loading of self-peptides, such allotype-specific properties may contribute to genotype-dependent differences in the immunopeptidome sampled by autoreactive CD8^+^ T cells.

Our β2-microglobulin perturbation experiments provide evidence of a neuron-enriched peptidome. We found that CRISPR/Cas9 deletion of β2M effectively produced a null HLA class I peptidome and we could reintroduce HLA class I expression specifically in iPSC-derived neurons. The detection of neurofilament light chain in serum and CSF is widely interpreted as a marker of neuronal and axonal injury [36–39]. This factor is elevated early in inflammatory demyelinating disease states and elevation correlates with clinical measures of disease severity and progression rate across numerous CNS diseases. Presentation of NEFL-derived peptides on HLA class I is consistent with both the presence of anti-neurofilament antibodies [40, 41] and neurofilament-reactive T cells [42, 43] in multiple disease states. Our findings identify NEFL-derived peptides as candidate neural antigens presented within the IFNγ-induced HLA class I ligandome, and we observed enhanced ex vivo T cell activation in response to NEFL protein in cultures derived from patients diagnosed with MS or GAD65-associated autoimmunity. The expression of CD69 marker was interpreted in conjunction with multiple orthogonal lines of evidence, including antigen-dependent downstream functional readouts of neuronal injury, which together support a biologically relevant T cell activation response rather than relying on CD69 as a standalone marker. In this system, activation was therefore defined functionally and in an antigen-dependent manner rather than solely on the basis of surface marker expansion.

The CEF-based system was designed as a proof-of-concept assay to determine whether IFNγ-treated human neurons are capable of presenting antigens sufficient for CD8^+^ T cell engagement. Because the system relies on forced expression of viral epitopes and expansion of pre-existing memory T cells, these experiments should not be interpreted as direct evidence for endogenous neuronal autoantigen-specific cytotoxicity. Several additional limitations constrain the interpretation of our findings. The wildtype HNAs are comprised of mixed neural lineages, so the immunopeptidomes identified in this study must be interpreted as a composite neural HLA class I ligandome. This motivated our use of the neuron-restricted β2M rescue to identify a more neuron-enriched immunopeptidome, but this approach also was limited by a much smaller peptide repertoire. While this is likely reflective of true limits on the extent of neuron presentation, it does confound the precision in our locus usage analysis. Likewise, inference of allele restriction from prediction and motif deconvolution is certainly informative, but it is not equivalent to allotype-specific immunoprecipitation approaches and quantitative analysis of HLA-A/B/C abundance. The pan-HLA class I antibody W6/32 may also recover non-classical HLA class I complexes, including HLA-E-associated ligands. Leader-sequence-derived peptides consistent with canonical HLA-E presentation were removed during preprocessing prior to downstream NetMHCpan analyses; however, inference of allele restriction from prediction algorithms remains an indirect approach and should be interpreted cautiously. Additionally, the relatively small number of donors limits generalizability of the observed HLA binding patterns. Nonetheless, our platform and the identification of a reference neuroimmunopeptidome provides a path for future work aimed at establishing the pathogenic significance of these candidate neuronal antigens, and whether these peptides are recognized by autoreactive T cells in vivo remains to be determined. Further deep immunopeptidomic discovery using our platform can be used to nominate both population and individual patient proteins for testing as CD8^+^ T cell targets in our co-culture system. Additional studies will be required to determine whether NEFL-derived peptides are directly recognized by autoreactive T cells in vivo or mediate pathogenic cytotoxicity.

## Materials and methods

### Human specimens

All human samples were obtained from individuals providing written informed consent following protocols approved by the Mayo Clinic Institutional Review Board (IRB 17-004547; IRB 13-007298), the Mayo Clinic Biospecimens Committee, and the Mayo Clinic Stem Cell Research Oversight Subcommittee. The induced pluripotent stem cell-derived human neural aggregates used for snRNAseq analysis were from a healthy donor (54-year-old white, non-Hispanic female). The iPSC-derived human neural aggregates and autologous CD8^+^ T cells used for the CEF experiments were from two healthy donors (31-year-old white, non-Hispanic female; 34-year-old white, non-Hispanic male). The induced pluripotent stem cell lines used for deep immunopeptidomics were: donor 1 = 73-year-old white, non-Hispanic female; donor 2 = 74-year-old white, non-Hispanic male; donor 3 = 56-year-old white, non-Hispanic female; donor 4 = 34-year-old white, non-Hispanic male, whose HLA class I haplotypes are indicated in the Supplemental Table 1. The samples used for the Antigen-Induced Marker assay included cells from 3 patients diagnosed with multiple sclerosis who had not received anti-CD20 therapy, 3 patients diagnosed with GAD65-associated CNS autoimmunity, and from 3 healthy controls.

### Induced pluripotent stem cell cultures and neural differentiation

Induced pluripotent stem cell clones were generated using Sendai virus reprogramming (CytoTune 2.0, Thermo Fisher) of adult dermal fibroblasts derived from skin punch biopsies. Trilineage potential, pluripotency, and sterility were assessed by the Mayo Clinic Biotrust and the Mayo Clinic Stem Cell and Organoid Core Facility. Induced pluripotent stem cells were differentiated into neural stem cells using dual SMAD inhibition and STEMdiff Neural Induction Medium (Stem Cell Technologies). Following neural induction, neural stem cells were cultured in neural stem cell expansion media consisting of KO DMEM/F-12, 1% Glutamax, 2% StemPro Neural Supplement (Thermo Fisher), 1% Anti‐Anti (Thermo Fisher) and 20 ng/mL FGFb (PreproTech) and EGF (Invitrogen). Induced pluripotent stem cells and neural stem cells were supplemented with 5 µM Rock Inhibitor (Y27632, Stemcell Technologies) for the first 24 h after thawing. Human neural aggregates were differentiated in media composed of KO DMEM/F‐12, 2% B27 supplement (Thermo Fisher), 1% Glutamax, 100 U/mL penicillin, 100 µg/mL streptomycin, 100 µM dibutyryl cyclic‐adenosine monophosphate (cAMP; Sigma), 5 µg/mL plasmocin (Invivogen), 1 mM sodium pyruvate, and 10 ng/mL each of BDNF (PreproTech), IGF1 (Corning) and GDNF (PeproTech) for at least 2 weeks, as previously described [15].

### CRISPR/Cas9-mediated knockout of B2M in induced pluripotent stem cell-derived neural stem cells

Human induced pluripotent stem cell-derived neural stem cells were engineered to disrupt expression of beta-2 microglobulin (*B2M*) using CRISPR/Cas9 genome editing. Synthetic single-guide RNAs (sgRNAs) targeting *B2M* were designed and synthesized by Synthego (now EditCo). Three sgRNAs targeting coding regions of *B2M* were used to maximize editing efficiency and promote frameshift-inducing insertions/deletions (indels): g1: CGGAGCGAGAGAGCACAGCG (PAM: AGG); g2: GGCCGAGATGTCTCGCTCCG (PAM: TGG); g3: ACTCACGCTGGATAGCCTCC (PAM: AGG). CRISPR ribonucleoprotein (RNP) complexes were generated by complexing recombinant Streptococcus pyogenes Cas9 nuclease with chemically modified sgRNAs according to the manufacturer’s recommendations. Neural stem cells were dissociated to a single-cell suspension and transfected with Cas9 RNP complexes using Lipofectamine CRISPRMAX. Following transfection, cells were recovered in pre-warmed neural stem cell maintenance medium and cultured under standard conditions. Genomic DNA was harvested several days after editing, and the targeted *B2M* loci were PCR amplified for Sanger sequencing analysis. Editing efficiency and indel profiles were quantified using the Synthego Inference of CRISPR Edits (ICE) analysis platform. ICE analysis demonstrated high editing efficiency with an indel frequency of approximately 98%, an ICE knockout score of 98, and a model fit (R²) of 0.98. The predominant editing outcomes consisted of + 1 bp and − 1 bp indels, consistent with frameshift-mediated disruption of *B2M* coding sequence (Figure S2).

### T cell coculture and antigen-induced marker assay

Autologous cryopreserved human peripheral blood mononuclear cells prepared as previously described [44] were thawed, washed, and allowed to rest at 37 °C in RPMI supplemented with 10% heat-inactivated human serum. Monocytes were isolated using EasySep Human Monocyte Isolation Kits (StemCell Technologies) and differentiated into dendritic cells over 72 h in the presence of granulocyte-macrophage colony-stimulating factor (50 ng/mL) and interleukin-4 (20 ng/mL) as previously described [44]. CD3^+^ T cells were purified from peripheral blood mononuclear cells using EasySep Release Human CD3 Positive Selection Kits (StemCell Technologies) and rested for 72 h in interleukin-7 (10 ng/mL).

Dendritic cells were pulsed with either CEF peptide pools (10 µg/mL) or full-length recombinant human neurofilament light chain protein (R&D Systems, Cat# 9715-NF-050; 10 µg/mL) and cocultured with autologous CD3^+^ T cells for 72 h in 96-well plates. Cells were then analyzed by flow cytometry (Attune NxT) using antibodies against CD3 (clone HIT3a), CD4 (clone OKT4), CD8 (clone HIT8a), and CD69 (clone FN50) to assess T cell activation. Per-subject CD69^+^ frequencies were summarized as the median of three technical replicates per condition. Between-group comparison of background-corrected NEFL response (Δ NEFL) were performed by two-sided Mann-Whitney U test, pooling MS and GAD65 subjects into a single “autoimmune CNS” group (*n* = 6) for comparison against healthy controls (*n* = 3).

For coculture experiments, following stimulation with CEF peptide pools, CD8^+^ T cells were purified using EasySep Human CD8 + Isolation Kits and added to autologous human neural aggregates (5 × 10^^5^ T cells/well in 96-well plates) and cocultured for 72 h prior to flow cytometry assessment as described above.

Automated live-cell imaging and analysis were performed using an IncuCyte SX5 Green/Orange/NIR or IncuCyte SX3 Green/Red system (Sartorius) with the neurite analysis module (NeuroTrack, Sartorius). The coculture starting time point was used as the reference value for signal normalization in all experiments.

### Single-nucleus RNA sequencing and analysis

Human neural aggregates were treated with IFNγ for 0, 24, 48, or 72 h with matched untreated controls at each timepoint. Aggregates were processed for single-nucleus RNA sequencing using the 10x Genomics Chromium Flex Gene Expression platform with the Human Whole Transcriptome probe set (Chromium_Human_Transcriptome_Probe_Set_v1.0.1_GRCh38-2020-A). Dounce homogenization was performed on ice in nuclei isolation buffer containing Tris-HCl (pH 7.4), NaCl, MgCl₂, Nonidet P-40, and DTT. Homogenate was filtered through a 40 μm cell strainer to remove debris and centrifuged at 500 × g for 10 min at 4 °C to pellet the nuclei. Nuclei were resuspended in PBS supplemented with 1% BSA and RNase inhibitor, stained with trypan blue, counted using an automated cell counter, and adjusted to a final concentration of approximately 3,500 nuclei/µL. Each sample was probed with one of four probe barcodes (BC001, BC002, BC003, BC004) and the probed samples were combined into two libraries (one of each probe per sample), taking 20,000 nuclei per probe barcode for an expected total of 80,000 nuclei per sample. Single-nucleus suspension samples were loaded onto a Chromium GEM-X FLEX Gene Expression Chip targeting recovery of 80,000 nuclei per sample. Nuclei were partitioned into Gel Bead-in-Emulsions (GEMs) using the Chromium Controller. Within each GEM, polyadenylated RNA transcripts were reverse transcribed using barcoded oligonucleotides containing a cell-specific barcode and unique molecular identifier (UMI). Following reverse transcription, GEMs were broken, and barcoded cDNA was recovered and amplified by PCR. Amplified cDNA was purified using SPRIselect magnetic beads. Sequencing libraries were generated by enzymatic fragmentation, end repair, A-tailing, adaptor ligation, and sample index PCR according to the manufacturer’s protocol. Library quality and fragment size distribution were assessed using a TapeStation, and libraries were quantified by fluorometric and qPCR-based methods. Libraries were sequenced on an Illumina platform using paired-end sequencing with a 28-bp read for cell barcode and UMI capture and a 90-100-bp read for transcript sequence. Sequencing depth targeted approximately 30,000–50,000 read pairs per nucleus. Reads were aligned and demultiplexed using Cell Ranger v9.0.0 against the GRCh38-2020-A reference.

Per-sample raw counts were loaded into Python and concatenated using scanpy v1.11.5 and anndata v0.11. Nuclei were retained if they had ≥ 1,500 detected genes, ≥ 2,000 total UMIs, and ≤ 5% mitochondrial reads. Doublet detection was performed using Scrublet (sc.pp.scrublet) with batch_key = library and expected_doublet_rate = 0.08; predicted doublets were removed. After filtering, 96,229 of 112,618 nuclei (85.4%) were retained, with 16,287 of 18,129 genes meeting a min_nuclei = 10 expression threshold. Ambient RNA contamination was estimated and corrected per sample using CellBender v0.3.0 with the --cuda flag, --total-droplets-included = 30,000, --fpr = 0.01, --epochs = 150, and --learning-rate = 1e-4. The MCKP-corrected output (cellbender_output_filtered.h5) for each sample was concatenated and aligned to the QC-filtered cell set, yielding a CellBender-corrected expression matrix of 96,229 cells × 16,287 genes with the original raw counts retained as a layer for sensitivity analysis. CellBender-corrected counts were preserved as a counts layer. The expression matrix was then total-count normalized to 10,000 UMIs per cell and log1p-transformed for downstream visualization and module scoring. Highly variable genes (*n* = 3,000) were selected using sc.pp.highly_variable_genes with flavor = ‘seurat_v3’, layer = ‘counts’, and batch_key = ‘library’ to account for library-level batch effects. Nuclei were integrated across libraries using scVI (scvi-tools v1.4.1) trained on the 3,000 highly variable genes with library as the categorical batch covariate and pct_counts_mt and n_genes_by_counts as continuous covariates. The model used a zero-inflated negative binomial gene likelihood with two encoder/decoder layers, 128 hidden units, and a 30-dimensional latent space. Training was performed on an NVIDIA RTX 4070 Ti SUPER GPU with max_epochs = 200, batch_size = 256, train_size = 0.9, and early stopping with patience = 10 epochs on validation ELBO; training converged at epoch 87. The 30-dimensional scVI latent representation was used to compute a 15-nearest-neighbor graph (sc.pp.neighbors) and a 2D UMAP embedding (sc.tl.umap, min_dist = 0.3).

To avoid clustering artifacts driven by the strong IFNγ-induced transcriptional response, cell types were assigned by direct marker expression rather than by Leiden clustering. Module scores were computed using sc.tl.score_genes for the following gene sets: pan-neuronal (MAP2, MAPT, NEFL, NEFM, STMN2, SYP, SYN1), glutamatergic (SLC17A7, SLC17A6, GRIN1, GRIN2B, CAMK2A, NEUROD2, NEUROD6), GABAergic (GAD1, GAD2, SLC32A1, DLX1, DLX2, DLX5), neural progenitor (SOX2, NES, VIM, PAX6, FABP7, HES1, HES5), and the antigen presentation module described below. Nuclei were assigned hierarchically: nuclei with neural progenitor score > 0.2 and pan-neuronal score ≤ 0 were labeled progenitors; nuclei with pan-neuronal score > 0 and progenitor score ≤ 0.2 were labeled neurons; nuclei with GABAergic score > 0.3 within the neuronal set were labeled GABAergic, those with glutamatergic score > 0.15 (and not GABAergic) were labeled glutamatergic, and remaining neurons were labeled “other neuron”; nuclei positive for both neuronal and progenitor markers were labeled transitional. Cell-type proportions were stable across IFNγ status (e.g., 22.3% glutamatergic in IFN-γ-treated versus 22.9% in untreated samples), confirming that the marker-based classification was independent of treatment condition.

An antigen presentation module score was computed per nucleus across all neurons using sc.tl.score_genes with the following genes: TAP1, TAP2, TAPBP, B2M, PSMB8, PSMB9, PSMB10, PSME1, ERAP1, ERAP2, NLRC5, IRF1, STAT1. The score represents the mean log-normalized expression of these genes minus the mean expression of a randomly selected size-matched control gene set drawn from genes with similar expression magnitude, providing a relative enrichment score that controls for differences in cellular sequencing depth and overall gene expression. Per-condition summary statistics (means, medians, distributions) were computed across all neurons (*n* = 40,780; pooled glutamatergic, GABAergic, and other neuronal subtypes). Violin plots were generated using seaborn v0.13. Heatmaps and cumulative distribution plots were generated using matplotlib v3.8. Figure panels were exported as SVG with editable text for downstream layout in Adobe Illustrator. Analyses were performed in Python 3.11 on Ubuntu 24.04 with key packages: scanpy v1.11.5, anndata v0.11, scvi-tools v1.4.1, PyTorch v2.6.0 + cu124 (CUDA 12.4), scikit-learn, scipy, numpy, pandas, matplotlib, and seaborn. CellBender v0.3.0 was run in a separate conda environment with PyTorch v2.3.1 + cu121 and pyro v1.8.6. All analyses were conducted on a single workstation with an NVIDIA RTX 4070 Ti SUPER GPU. Code and analysis scripts are available upon request.

IFNγ receptor subunit expression and co-expression analysis were performed on unstimulated cells pooled across all four timepoints (*n* = 49,643 cells, ifng = ‘minus’ condition). Cell type labels were transferred from the HNA integration reference atlas (146,597 nuclei, 23 clusters) using sc.tl.ingest (scanpy v1.11.5), which projects query cells into a shared PCA space computed on the 16,287 genes common to both datasets and assigns labels by k-nearest neighbor (k = 15) in that space. The reference atlas PCA was recomputed on 2,000 highly variable genes (Seurat flavor, 50 components) after gene intersection, with a 30-nearest-neighbor graph used for the neighbors step. Transferred labels (23 categories) were consolidated into five groups for visualization: Astrocytes, Projection neurons, Excitatory neurons (Brainstem/spinal glutamatergic VGLUT2+, glutamatergic immature, Dorsal spinal cord ZIC+/HOX-C/A 4–9+, Hindbrain/spinal LHX1/LBX1+, Raphe serotonergic), Inhibitory neurons (Hindbrain/spinal GABAergic), and Immature neurons (Neurogenic/immature, Maturing PTPRZ1+).

Dual-positive cells were defined as nuclei with log-normalized IFNGR1 > 0 and IFNGR2 > 0. Spatial enrichment of dual-positive cells within each consolidated category was quantified using a k-nearest-neighbor (k = 50) enrichment index computed in UMAP space: for each cell, the fraction of its 50 within-category nearest neighbors that were dual-positive was computed; the enrichment ratio is the mean local fraction in dual-positive cells divided by the global dual-positive fraction for that category. Chi-square tests assessed significance of spatial clustering. Excitatory neuron sub-clustering was performed on all 40,566 neurons (all conditions) using the 30-dimensional scVI latent representation with a 15-nearest-neighbor graph. Leiden clustering was run at resolutions 0.2, 0.3, and 0.5; resolution 0.2 (five clusters) was selected by maximum interquartile range of dual-positive fraction across clusters. Subclusters were classified as dual-positive enriched (enrichment ratio ≥ 1.3; *n* = 13,124 cells) or not-enriched (all remaining subclusters). An AP class I score was computed per nucleus for the excitatory neuron subset using the following genes: TAP1, TAP2, TAPBP, B2M, PSMB8, PSMB9, PSMB10, PSME1, PSME2, ERAP1, ERAP2. Group comparisons used the Mann-Whitney U test. Per-gene log2 fold-change between dp-enriched and pooled not-enriched subclusters was computed from mean log-normalized expression at 24 h IFNγ+; the not-enriched reference was the weighted mean of dp-depleted (*n* = 78) and intermediate (*n* = 2,693) subclusters, with a pseudocount of 0.01. Note that the 10x Flex Human probe panel does not include HLA-A, HLA-B, or HLA-C; B2M serves as a proxy for HLA class I complex formation in all snRNA-seq analyses.

### Crosslinking of W6/32 clone MHC Class I antibody to protein A-sepharose 4B beads

5 mg of antibody was loaded on 1 mL of Protein A-Sepharose 4B beads (Invitrogen) packed in a polypropylene column (BioRad) and incubated for 30 min at room temperature. Antibody-bound beads were washed with borate buffer (pH 9) and incubated with 20 mM dimethyl pimelimidate (DMP) linker for 45 min. Crosslinking was halted by incubating the column with ethanolamine for 2 h. The column was washed with PBS and stored in PBS containing 0.02% sodium azide at 4 °C. Prior to use, the column was washed with 0.1 N acetic acid and equilibrated with 100 mM Tris-HCl, pH 8.0.

### MHC-peptide complex enrichment

Human neural aggregates were treated with IFNγ (100 ng/mL) for 72 h. In some cases, 7–10 days prior to IFNγ treatment the mature aggregates were transduced with adeno associated viral vectors (AAV1-Syn1.hB2M/T2A/eGFP: oPRE, VectorBuilder; AAV1.Syn.eGFP: WPRE, AddGene 50465-AAV1; AAV1.Syn.sig-CEF-DC-LAMP-T2A-eGFP.WPRE, Vector Biolabs). To obtain sufficient protein lysates for immunopeptidomic analysis 120–200 million mature neural cells were used for each condition. After IFNγ treatment, cells were washed on ice with cold PBS. Flasks were immediately scraped and cells were pelleted by centrifugation, snap frozen in liquid nitrogen, and stored until further processing. MHC-peptide complexes were enriched as described [25]. Human neural aggregates were lysed in buffer containing 0.25% sodium deoxycholate, 0.2 mM IAA, 1 mM EDTA, 1 mM PMSF, 1% octyl-B-glucopyranoside, and 1:200 protease inhibitor cocktail for 1 h on ice. Cell debris was removed by centrifugation at 25,000 x g at 4 °C for 50 min. The lysate was loaded onto affinity columns containing W6/32 crosslinked to protein A beads and incubated for 1 h with gentle rotation. The column was washed sequentially with (1) 150 mM NaCl in 20 mM Tris-HCl pH 8.0, (2) 400 mM NaCl in 20 mM Tris-HCl pH 8.0, (3) 150 mM NaCl in 20 mM Tris-HCl pH 8.0, and (4) 20 mM Tris-HCl pH 8.0. MHC-bound peptide complexes were eluted using 1% TFA and the eluate was loaded on Sep-Pak C18 columns to purify the eluted peptides. The peptides were dried using a Speedvac concentrator prior to LC-MS/MS analysis.

### LC-MS/MS analysis

LC-MS/MS analysis was carried out on an Orbitrap Eclipse Tribrid mass spectrometer (Thermo Scientific) connected online to a Dionex RSLC3000 liquid chromatography system (Thermo Scientific). Peptides were suspended in solvent A (0.1% formic acid) and loaded on a trap column (PepMap C_18_ 2 cm × 100 μm, 100 Å) followed by high resolution separation column (EasySpray 50 cm X 75 μm, C_18_ 1.9 μm, 100 Å, Thermo Scientific). The mass spectrometer was operated in data dependent mode with a cycle time of 2 s. Survey MS scan was acquired in Orbitrap mass analyzer with 120 K resolution, 4xe5 AGC target and 50 ms injection time. Monoisotopic precursor ions with charge state 2–4 were subjected to MS/MS with top scan priority followed by precursor with charge 1. Precursor ions (z = 2–4) were fragmented with 28% HCD normalized collision energy and acquired in orbitrap mass analyzer with 15 K resolution. Precursor ions (z = 1) with a mass range of 700–1400 m/z were fragmented with 32% HCD normalized collision energy and analyzed in orbitrap analyzer. Dynamic exclusion was enabled with 30 s exclusion duration. Additional filters included monoisotopic precursor selection and intensity threshold of 2.5 × 10^4^.

For reproducibility assessment, replicate induced pluripotent stem cell-derived neural stem cell cultures from the same donor were independently processed and analyzed at the University of Arizona and Mayo Clinic Rochester proteomic core facilities, and overlap in recovered peptides/proteins and inferred HLA-binding motifs was compared.

### Immunofluorescence and confocal microscopy

Human neural aggregates were initially fixed by adding an equal volume of 4% paraformaldehyde (PFA) prepared in 0.1 M phosphate buffer (pH 7.4) directly to the culture medium and incubating for 25 min at room temperature. The medium was aspirated and replaced with ice-cold 4% PFA in phosphate buffer for another 25 min. Cells were permeabilized in 0.1% Triton X-100 in PBS and blocked for at least 30 min in PBS containing 5% normal donkey serum, 1% bovine serum albumin, 0.01% sodium azide, and 0.1% Triton X-100. Cells were subsequently incubated overnight at 4 °C with primary antibodies (anti-HLA-ABC (W6/32; Thermo Fisher), anti-GFAP (2.2B10; Thermo Fisher), anti-MAP2 (AP20; Millipore Sigma), anti-neuron-specific beta-III tubulin (MAB1195; R&D Systems)) diluted to 5 µg/mL in blocking buffer. The following day, cells were washed three times with 0.1% Triton X-100 in PBS and incubated for 2 h at room temperature with secondary antibodies diluted 1:200 in blocking buffer. After washing, nuclei were counterstained with DAPI prior to imaging. Images were acquired using an Olympus confocal microscope and post-processed in ImageJ and Photoshop.

### Western blot

Protein samples were prepared in urea-based sample buffer (8 M urea, 50 mM Tris-HCl pH 7.5, 75 mM NaCl) supplemented with protease and phosphatase inhibitors. Samples were mixed with SDS-containing loading buffer supplemented with 50 mM DTT and resolved by SDS-PAGE on 4–12% gradient gels. Proteins were transferred onto 0.2 μm nitrocellulose membranes using wet transfer at 100 V for 90 min. Membranes were briefly stained with Ponceau S to confirm equal transfer and loading, then blocked in 5% BSA in TBS-T (Tris-buffered saline, 0.1% Tween-20) for 1 h at room temperature. Membranes were incubated overnight at 4 °C with primary antibodies (anti-HLA-ABC (W6/32; Thermo Fisher), anti-HLA-A (EP1395Y; Abcam), anti-HLA-B (23GB6375; Thermo Fisher), anti-HLA-C (EPR6749; Abcam)) diluted in blocking buffer, washed three times in TBS-T, and incubated for 1 h at room temperature with HRP-conjugated secondary antibodies. Immunoreactive bands were detected using enhanced chemiluminescence (ECL) substrate and imaged with a ChemiDoc imaging system (Bio-Rad). Band intensities were quantified using Image Lab software (Bio-Rad).

## Supplementary Information

Supplementary Material 1.

## Data Availability

The datasets generated and/or analyzed during the current study are available from the corresponding author on reasonable request. Data are not yet publicly available because they are being prepared for deposition in appropriate public repositories; accession numbers/DOIs will be provided upon publication. Requests that involve potentially identifiable donor-level information will require an appropriate data use agreement and applicable approvals.
