# Supplementary material for: Human iPSC-derived neurons stimulated with IFNγ present HLA class I-restricted antigens to cytotoxic CD8+ T cells
Source: J Neuroinflammation. 2026 Jun 17;23:285. doi: 10.1186/s12974-026-03915-y (PMC13508380; doi:10.1186/s12974-026-03915-y)
Supplement: Supplementary file 1 [file 12974_2026_3915_MOESM1_ESM.pdf]

Figure S1. Cross-laboratory reproducibility of iPSC-derived neural stem cell immunopeptidomics.
(A) Immunopeptidomes from replicate iPSC-derived neural stem cell cultures generated from the same donor were independently analyzed by IP-LC-MS/MS at the University of Arizona and Mayo Clinic Rochester proteomic core facilities. Venn diagrams show overlap of recovered peptides and source proteins between replicate datasets. **(B)** GibbsCluster analysis of replicate 1 and replicate 2 datasets demonstrating consistent HLA-binding sequence motifs across samples. Sequence logos corresponding to predicted HLA allele-specific binding motifs are shown.

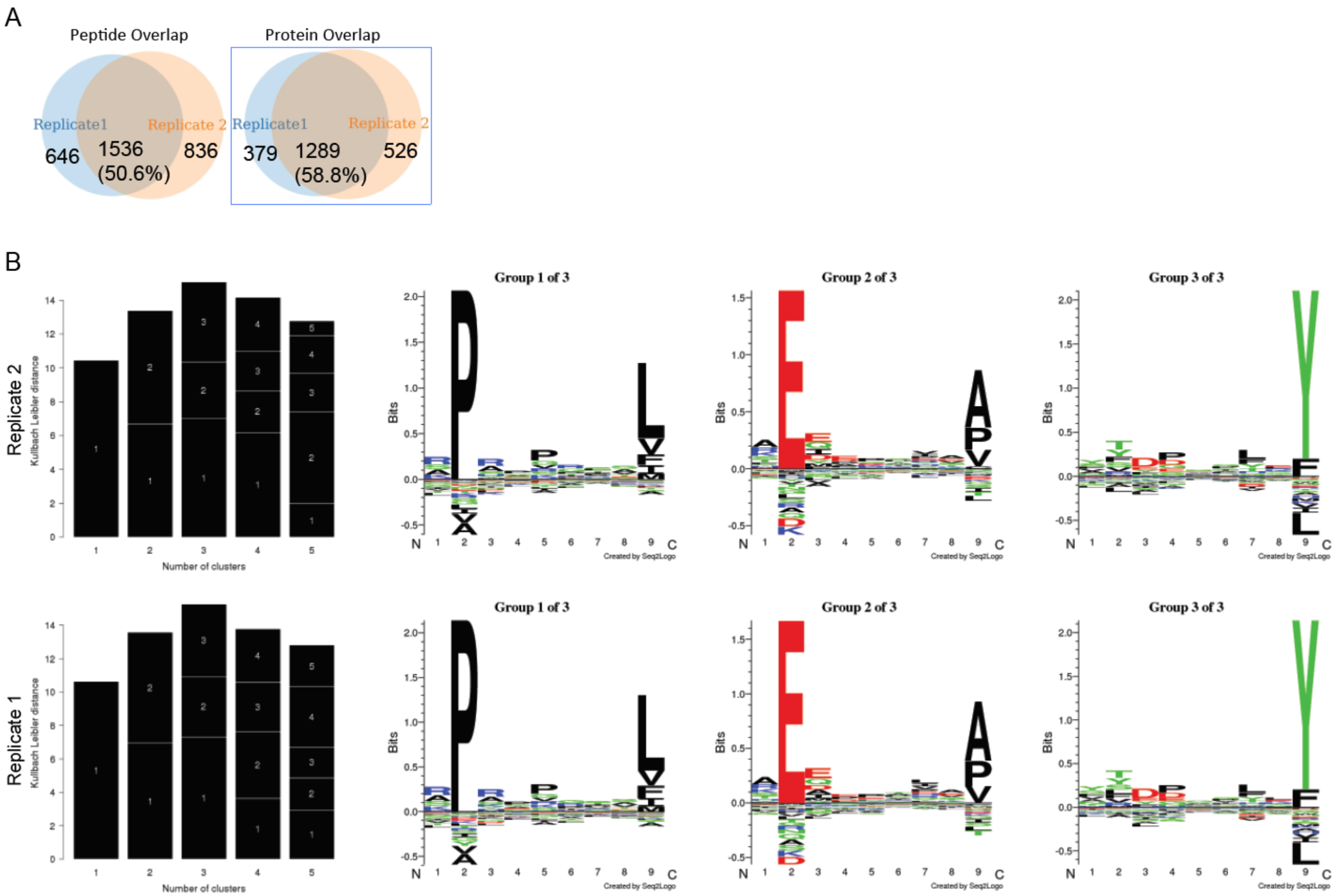

Figure S2. CRISPR/Cas9-mediated knockout of B2M in iPSC-derived neural stem cells.
(A) Synthego ICE analysis of genomic DNA from edited NSCs following CRISPR/Cas9 ribonucleoprotein (RNP) delivery targeting the *B2M* locus using three sgRNAs. ICE deconvolution demonstrated successful editing with an estimated indel frequency of 98%, model fit (R^2) of 0.98, and knockout score of 98. Predominant editing outcomes consisted of +1 bp and −1 bp indels, each contributing ~49% of the edited alleles. PAM sequences and guide target sequences are shown. **(B)** Distribution of indel sizes detected at the targeted *B2M* locus. The edited sample exhibited highly enriched indels centered around single base pair insertions and deletions relative to the unedited control. Discordance traces across the Sanger sequencing window demonstrate robust editing localized to the sgRNA target regions. **(C)** Representative Sanger sequencing chromatograms from edited and control samples across the three sgRNA target sites (G1–G3). Edited samples displayed mixed sequence traces and loss of signal phasing beginning at the predicted Cas9 cleavage sites, consistent with high-efficiency indel formation, whereas control samples retained clean wild-type sequence profiles. *B2M*-knockout NSCs were subsequently differentiated into neurons and used for immunopeptidomic studies comparing AAV1.Syn-B2M rescue versus empty vector conditions.

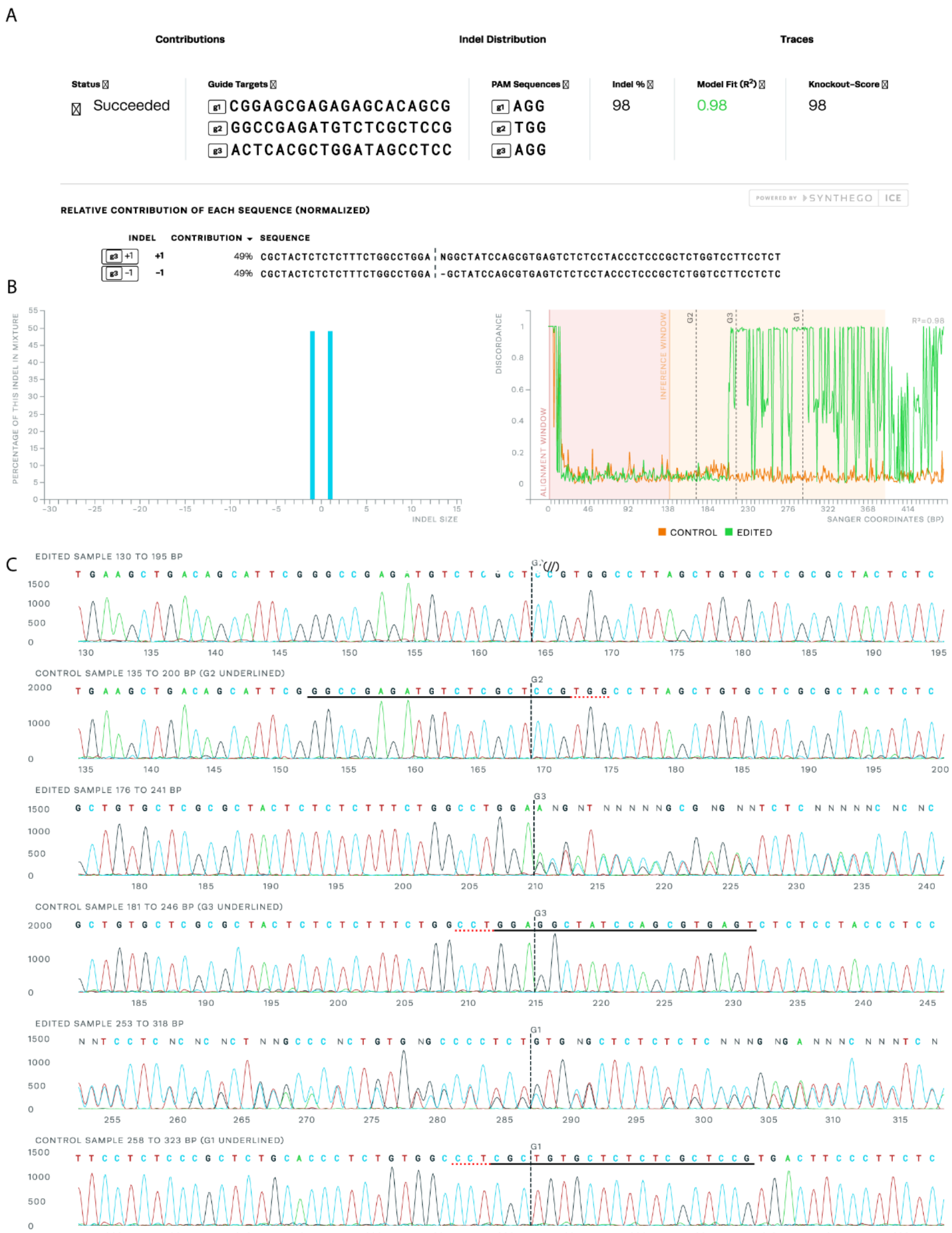

Supplemental Figure 3. Antigen presentation kinetics in HNA neurons and IFNγ receptor co-expression in excitatory neurons.

(**A**) Cumulative distribution of antigen presentation module scores across neurons in each condition. Each curve plots the percentage of neurons with a module score exceeding the value on the x-axis. The four untreated curves (blues) and the IFNγ-treated 0 h curve cluster tightly together, confirming a stable baseline. The 24, 48, and 72 h IFNγ-treated curves (oranges) overlay each other, demonstrating that the response reaches a plateau by 24 hours and remains stable thereafter. (**B**) Mean log-normalized expression of individual antigen presentation genes by condition and time across all neurons. Each row is one gene. (**C**) Full platform UMAP (n = 96,229 cells) showing excitatory neurons colored by IFNGR dual-positive status (red = IFNGR1+ and IFNGR2+; n = 17,163; grey = non-dual-positive; n = 23,403). All other cells shown in light grey background. (**D**) Within-excitatory-neuron UMAP showing IFNGR dual-positive class assignment (dp-enriched red; dp-depleted blue; intermediate orange). (**E**) Within-excitatory-neuron UMAP (n = 40,566 cells; Leiden resolution 0.2) showing transferred subtype labels (Glut. VGLUT2+ grey; Glut. immature light grey; Dorsal SC ZIC+ red; LHX1/LBX1+ blue; Raphe 5-HT green). Dorsal SC (ZIC+) neurons correspond spatially to the dp-enriched class shown in (**D**).

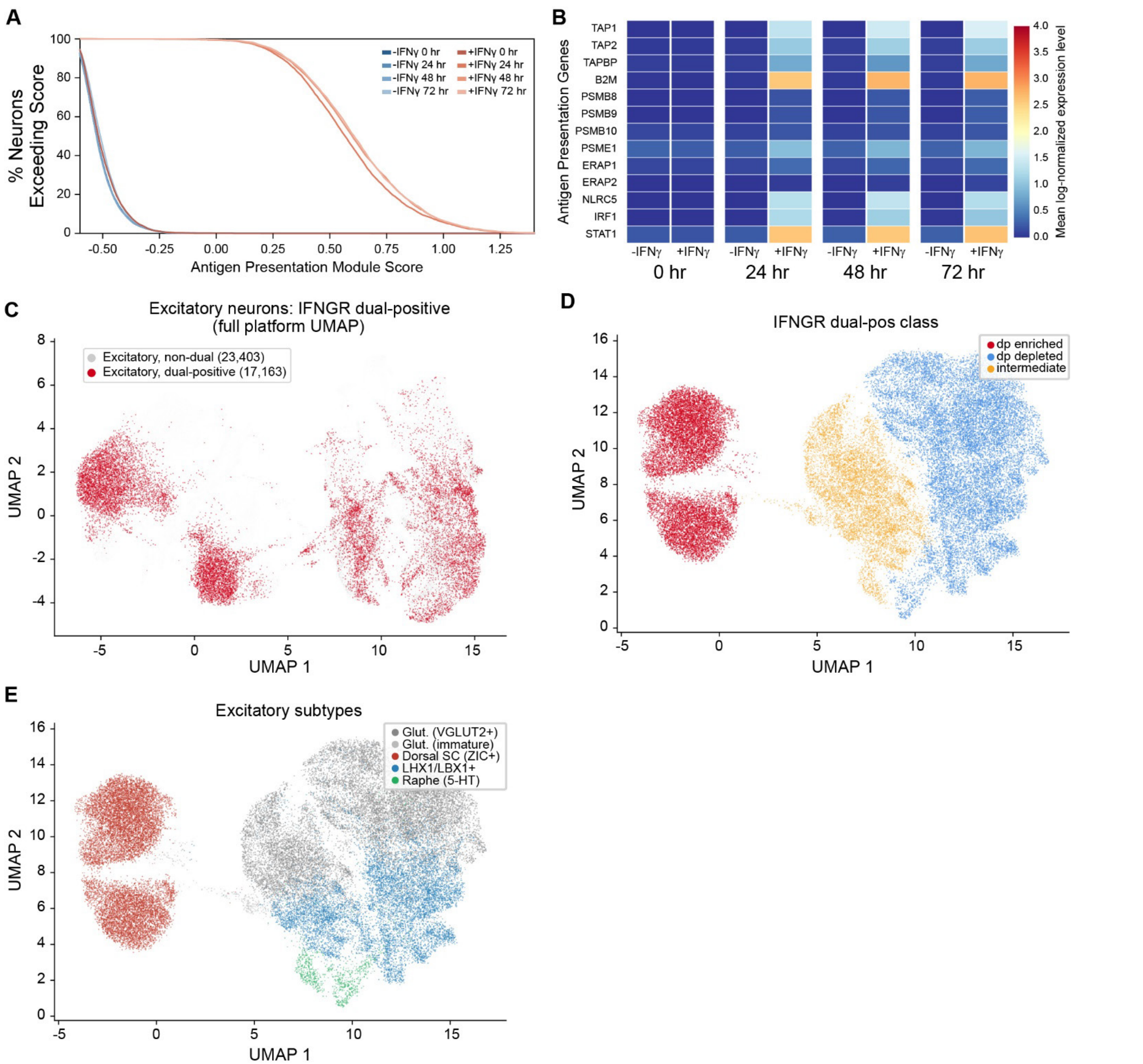

Supplemental Table 1

	HLA-A	HLA-A	HLA-B	HLA-B	HLA-C	HLA-C
Donor 1	A*02:01:01	A*03:01:01	B*07:02:01	B*51:01:01	C*07:02:01	C*14:02:01
Donor 2	A*02:01:01	A*29:02:01	B*40:01:02	B*44:04	C*03:04:01	C*16:01:01
Donor 3	A*03:01:01	A*24:02:01	B*15:01:01	B*44:03:01	C*03:04:01	C*16:01:01
Donor 4	A*01:01:01	A*31:01:02	B*08:01:01	B*44:03:01	C*04:01:01	C*07:01:01
